# Identification and function of ETH receptor networks in the silkworm *Bombyx mori*

**DOI:** 10.1038/s41598-021-91022-8

**Published:** 2021-06-03

**Authors:** Ivana Daubnerová, Ladislav Roller, Honoo Satake, Chen Zhang, Young-Joon Kim, Dušan Žitňan

**Affiliations:** 1 Institute of Zoology, Slovak Academy of Sciences, Dúbravská cesta 9, 84506 Bratislava, Slovakia; 2 Bioorganic Research Institute, Suntory Foundation for Life Sciences, Kyoto, Japan; 3 School of Life Sciences, Gwangju Institute of Science and Technology (GIST), Oryongdong, Buk-gu, Gwangju, 61005 Republic of Korea

**Keywords:** Physiology, Zoology

## Abstract

Insect ecdysis triggering hormones (ETHs) released from endocrine Inka cells act on specific neurons in the central nervous system (CNS) to activate the ecdysis sequence. These primary target neurons express distinct splicing variants of ETH receptor (ETHR-A or ETHR-B). Here, we characterized both ETHR subtypes in the moth *Bombyx mori *in vitro and mapped spatial and temporal distribution of their expression within the CNS and peripheral organs. In the CNS, we detected non-overlapping expression patterns of each receptor isoform which showed dramatic changes during metamorphosis. Most ETHR-A and a few ETHR-B neurons produce multiple neuropeptides which are downstream signals for the initiation or termination of various phases during the ecdysis sequence. We also described novel roles of different neuropeptides during these processes. Careful examination of peripheral organs revealed ETHRs expression in specific cells of the frontal ganglion (FG), corpora allata (CA), H-organ and Malpighian tubules prior to each ecdysis. These data indicate that PETH and ETH are multifunctional hormones that act via ETHR-A and ETHR-B to control various functions during the entire development—the ecdysis sequence and associated behaviors by the CNS and FG, JH synthesis by the CA, and possible activity of the H-organ and Malpighian tubules.

## Introduction

Ecdysozoa represents a very diverse and numerous group of invertebrate animals that have to regularly shed their exoskeleton (cuticle) to successfully proceed during the entire development. In most animals the mechanisms governing this intricate behavioral process called the ecdysis sequence have not been elucidated. So far, only a few insect and crustacean species have been used for detailed analysis of signaling pathways underlying this important behavior that enables the animal to enter the next developmental stage^1–4^. Various physiological and molecular approaches showed that Inka cells producing ETHs and central neurons expressing ETHRs are crucial components required for activation of the ecdysis sequence^5–9^. Identification of ETHs and their receptors in numerous insects, as well as several representatives of crustaceans and mites indicates that this signaling pathway may be conserved and widespread in many arthropods^10–13^. In all insects and other arthropods examined, two alternatively spliced receptor isoforms (ETHR-A and ETHR-B) are derived from a single *ethr* gene^11^. Although differential expression of each receptor isoform has been described in separate neuronal subsets in the CNS of the moth *Manduca sexta* and the fruitfly *Drosophila melanogaster*^7–9^, only several neurons have been identified and functionally characterized. Most ETHR-A and a few ETHR-B neurons produce numerous neuropeptides including kinins, CRF-like diuretic hormones (DHs), calcitonin-like DH31 (CT), allatostatin-A (AST-A), eclosion hormone (EH), FMRFamides, crustacean cardioactive peptide (CCAP), myoinhibitory peptides (MIPs), bursicon, neuropeptide F (NPF) and short neuropeptides F (sNPFs) Upon activation of ETHRs, these neuropeptides are released to control consecutive phases of the ecdysis sequence. In vitro experiments revealed that application of specific neuropeptides induced different pre-ecdysis or ecdysis motor programs in the isolated CNS of *M. sexta*^7,14,15^, or genetic manipulation of specific subsets of neurons expressing ETHR-A in *D. melanogaster* led to initiation or suppression of distinct phases of the ecdysis sequence^8,9,16–19^. These data demonstrated that kinins and DHs control pre-ecdysis, while a cascade of EH, CCAP, MIP and bursicon regulate ecdysis and post-ecdysis behaviors. However, roles of additional neuropeptides produced by ETHR neurons has not been examined^4^. Moreover, very little is known about functions and developmental changes of remaining non-peptidergic neurons expressing these receptors.

Increased ETHR levels in extracts of the corpora cardiaca—c. allata (CC-CA), epidermis, gut, Malpighian tubules and gonads of two diverse insect species^20,21^ indicate pleiotropic roles of ETHs during development. Indeed, recent papers showed that ETH signaling is essential for production of juvenile hormone (JH) and reproduction in mosquitoes and flies^22–24^. ETH action on another peripheral organ, the frontal ganglion (FG), may be associated with regulation of air swallowing behavior^25,26^. However, specific physiological or behavioral outcomes of ETH action on its receptors in other peripheral organs remain to be determined.

To better understand pleiotropic actions of ETH signaling, we mapped expression of ETHR-A and ETHR-B in the CNS and peripheral organs during development of *B. mori*. We identified key peptidergic ensembles of ETHR-A and ETHR-B neurons that show considerable changes during metamorphosis. We also demonstrate for the first time possible roles of pigment dispersing factor (PDF), allatostatin-CC (AST-CC) and sNPFs in the ecdysis sequence. Identification of several peripheral organs and cells expressing ETHRs indicates multiple additional physiological and behavioral functions of ETH, including air swallowing, water balance, reproduction and biosynthesis/release of various biologically active compounds. We believe our data shed more light on neuropeptide control of the initiation or termination of different phases of the ecdysis sequence and help to decipher additional roles of ETH signaling required for proper development.

## Results

### Organization and characterization of ETHR in *B. mori*

**Figure 1:**
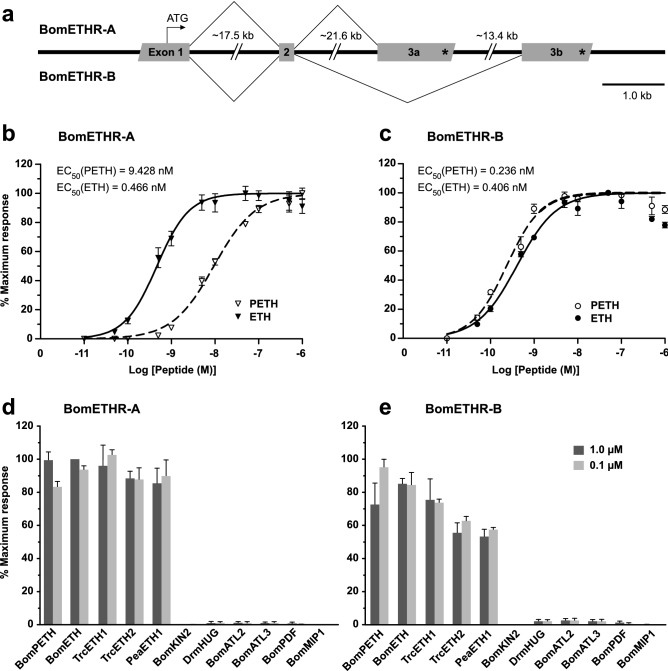
Genomic structure and characterization of ETHRs. (**a**) Schematic representation of *ethr* gene structure in *B. mori.* The receptor has two alternative transcripts with mutually alternative exons 3a and 3b. Exons and introns are indicated by grey boxes and solid lines, respectively. The stop codon is indicated by a star (*). (**b**, **c**) Dose–response curves for ETHR-A and ETHR-B heterologously expressed in CHO cells following application of different concentrations of PETH and ETH. Each response is expressed as a percentage of maximum peak luminescence induced by the respective ligand. Each data point is a mean value ± SE (n = 3). Insets show EC_50_ values for each ligand. (**d**, **e**) Luminescence produced by CHO cells expressing ETHR-A and ETHR-B after application of additional peptide ligands (0, 1 μM and 1 μM), normalized against the response to 1 μM ETH (**d**) and 0,1 μM PETH (**e**) respectively. Synthetic peptides used in the assay are listed in Supplementary Table S2 online. Bom, *Bombyx mori*; Trc, *Tribolium castaneum*; Pea*, Periplaneta Americana*; Drm, *Drosophila melanogaster*.

Two splice variants of the silkworm ETHR (referred as BNGR-A6-A and BNGR-A6-B) were first described in a comprehensive study focused on identification and expression analysis of the entire neuropeptide GPCR transcriptome in *B. mori*^20^. Using BNGR-A6-A and BNGR-A6-B transcript sequences to survey *B. mori* whole-genome database (Kaikobase;^27^) we found a single *ethr* gene located on chromosome 26 consisting of four exons and three introns. The first two exons are common for both receptor subtypes, whereas differential splicing of the last two exons produces ETHR-A or ETHR-B subtype with mutually alternative exons 3a and 3b (Fig. 1a). These exons encode amino acid sequences from the end of 4th transmembrane segment to the C-terminus, accounting for ∼60% of the receptor protein.

To determine specificity and potency of pre-ecdysis and ecdysis triggering hormones (PETH and ETH) from *B. mori* to activate identified receptors we transiently expressed DNA encoding either ETHR-A or ETHR-B in CHO cells and employed heterologous aequorin-based calcium mobilization assay. Both receptors responded to PETH and ETH in a dose–response manner, with ETHR-A showing much higher affinity to ETH (EC_50_ = 0,466 nM) than to PETH (EC_50_ = 9,428 nM) (Fig. 1b). These results correspond to differences in ETHR-A sensitivity observed in the previous study employing Ca^2+^ measurements using fura-2 in HEK293 cells^20^. CHO cells expressing the ETHR-B showed similar responses to PETH and ETH (Fig. 1c) with half maximal effective concentrations 0,236 nM and 0,406 nM, respectively. Neither of analyzed receptors was activated by other unrelated insect neuropeptides except for ETHs from the cockroach *Periplaneta americana* and the beetle *Tribolium castaneum*, confirming specificity of the ligand-receptor interaction (Fig. 1d, e). In addition, CHO-K1 cells transfected with empty pcDNA3.1 + vector showed no detectable response to any of the tested peptides (data not shown), demonstrating that the luminescence was a result of specific PETH or ETH binding to the transiently expressed ETHRs.

### ETHR-A expression in the CNS

**Figure 2:**
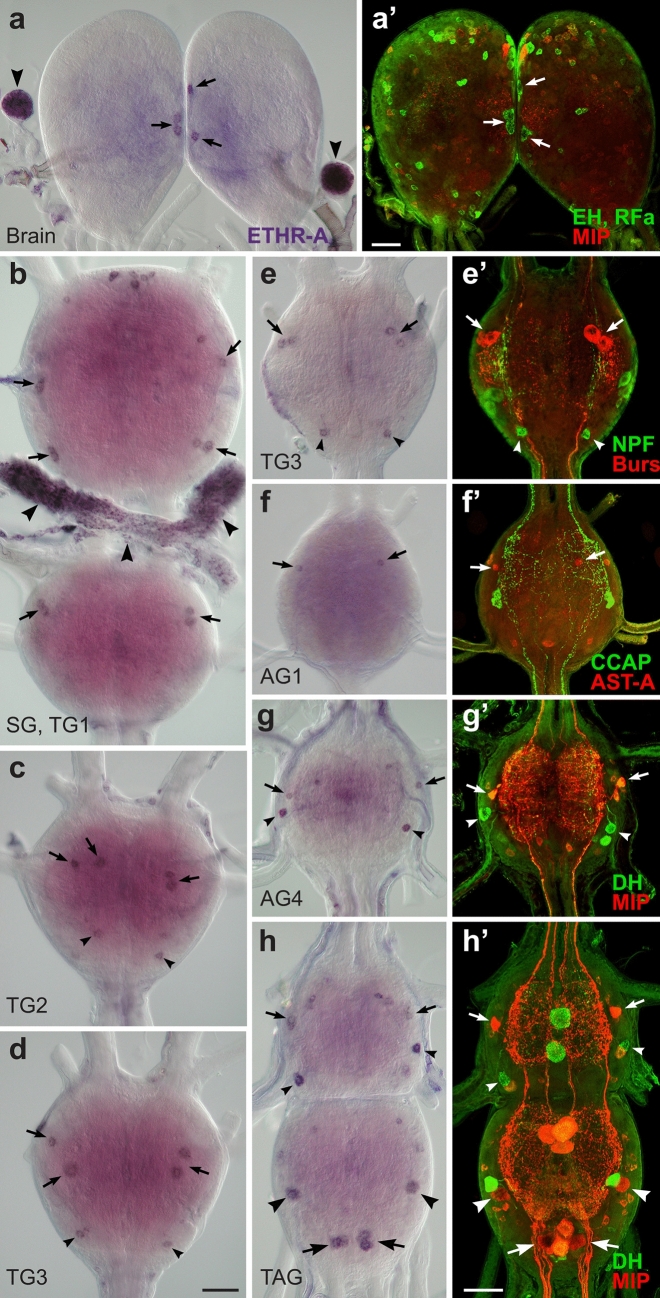
ETHR-A expression in the CNS of pharate larvae. (**a–h’**) ISH with a probe specific for ETHR-A transcript and subsequent staining of the same ganglia with various antibodies. (**a**, **a’**) Colocalization of ETHR-A and EH in two pairs of VM neurons in the brain (arrows; green) and strong ETHR-A signal in the CA (arrowheads). (**b–d**) ETHR-A expression in neurons 27/704 of the SG and TG1-3 (arrows), posterior DLT interneurons in the TG2-3 (small arrowheads) and in the H-organ (large arrowheads). (**e**, **e’**) Colocalization of ETHR-A and bursicon in cells 27/704 (arrows; red) and a pair of posterior DLT neurons producing NPF in the TG3 (arrowheads; green). (**f**, **f’**) A pair of small neurons in anterior part of the AG1 expressing ETHR-A and AST-A (red; arrows). (**g–h’**) Examples of ETHR-A colocalization with DHs in L_3,4_ neurons (small arrowheads; green) and with MIPs in IN-704 (small arrows; red) of the AG4 and AG7 which is anterior neuromere of the TAG. (**h**, **h´**) Identification of lateral VL8 (large arrowheads; red) and medial PM9 neurons (large arrows; red) coexpressing ETHR-A and MIP in the posterior TAG. Scale bars = 50 μm.

**Figure 3:**
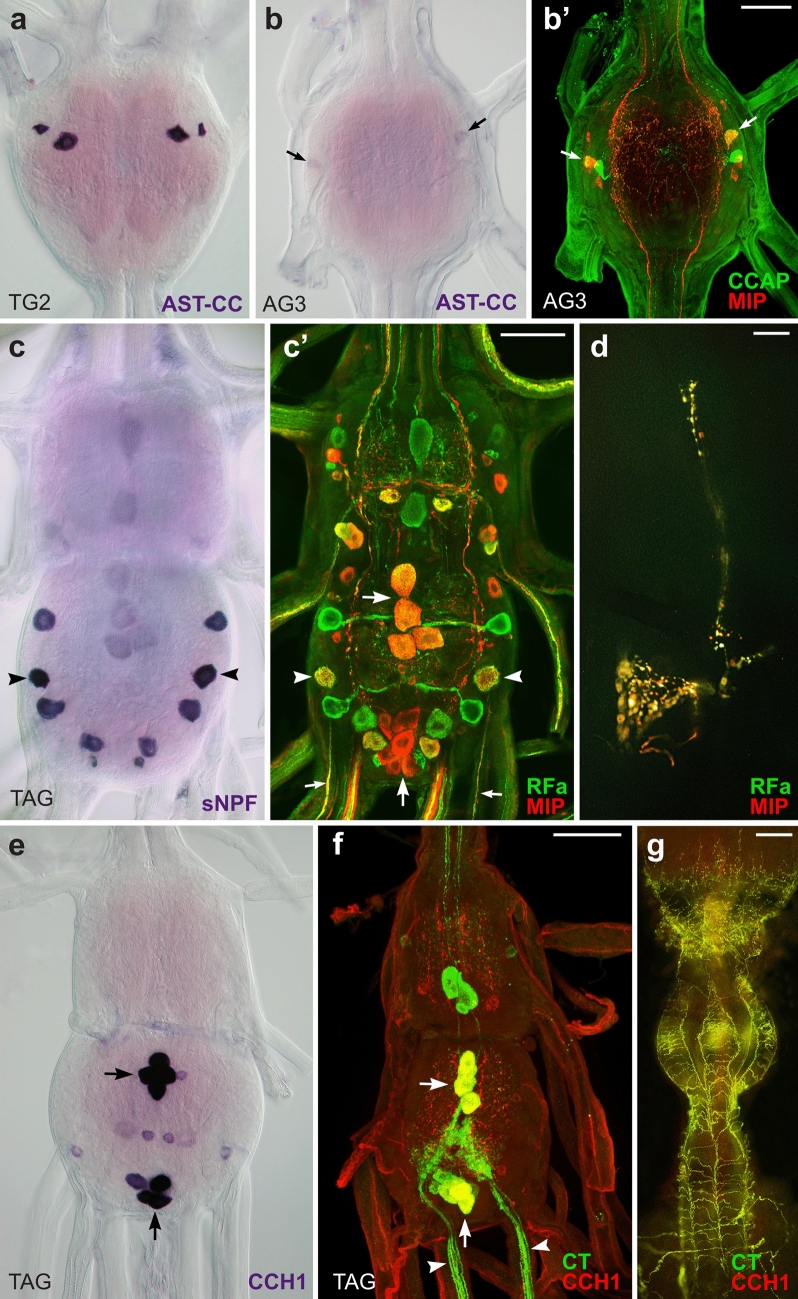
Identification of peptidergic neurons and their projections in the larval CNS. (**a**) Strong expression of AST-CC in cells 27/704 of the TG2 detected by ISH. (**b**, **b´**) Low levels of AST-CC transcript in IN-704 neurons of AG3 identified with antibodies to CCAP and MIPs (arrows; yellow). (**c**, **c’**) Lateral VL8 neurons in the TAG (arrowheads; yellow) identified by ISH with sNPF probe followed by double staining with antibodies to MIP (red) and RFamide (green). VL8 axons project via ventral nerves (small arrows; yellow) and arborize on muscle surface of 9th segment (**d**). Numerous varicosities in axon terminals indicate neuropeptide release from these putative neurohemal sites. (**e**) Medial PM8 and PM9 neurons detected by ISH with CCH1 probe (arrows). (**f**) Antibody staining confirmed colocalization of CCH1 (red) and CT (green) in PM8 and PM9 neurons (arrows; yellow) that project axons via proctodeal nerves (arrowheads) and (**g**) innervate the hindgut. These medial neurons are also stained with MIP antibody (**c’**; large arrows; red). Scale bars a-c´, e, f = 50 µm, d = 25 µm, g = 300 µm.

To localize and identify central neurons expressing ETHRs, we employed in situ hybridization (ISH) with probes specific for each receptor isoform followed by immunohistochemical staining (IHC) with antibodies against different neuropeptides (see Supplementary Table S3 online). In pharate larvae ETHR-A was expressed in numerous neurons producing various neuropeptides including EH, bursicon, AST-A, allatostatin-CC (AST-CC), CCAP, MIPs, kinins, DHs, CT, NPF, sNPFs and CCHamide 1 (CCH1) (Figs. 2, 3). Consistent ETHR-A expression was detected in ventromedial neurosecretory cells containing EH (Fig. 2a, a’), 3–4 small lateral neurons in the brain and 4–5 small weakly labelled neurons in the frontal ganglion. ETHR-A probe also labelled a cluster of 6–8 small neurons in the anterior SG, dorsolateral neurosecretory cells NS-27 and interneurons IN-704 (cells 27/704) coexpressing bursicon and AST-CC in the SG and TG1-3 and posterior dorsolateral neurons producing NPF in the TG1-3 (designated here as DLT1-3) (Fig. 2b–e). Interestingly, very strong ETHR-A expression was observed in endocrine cells of the CA (Fig. 2a) and small elongated cells in the H-organ (Fig. 2b). In the abdominal ganglia 1–8 (AG1-8) ETHR-A transcript was detected in a pair of small anterior neurons producing AST-A (Fig. 2f, f’). In the AG2-7 ETHR-A was colocalized with AST-CC, CCAP and MIPs in paired dorsolateral interneurons 704 (IN-704) and kinins and DHs in neurosecretory cells L_2,3_ (Fig. 2g–h’). In the posterior terminal abdominal ganglion (TAG) which is composed of fused neuromeres AG8-10, ETHR-A probe labelled lateral neurons VL8 producing sNPFs and MIPs, and 4–6 posteromedial PM9 neurons containing CT, MIPs and CCH1 (Fig. 2h, h’;^28^).

A neuropeptide AST-CC is a paralog of AST-C that has been identified in silico in genomes of numerous insects^29^. So far, its function and cellular localization have not been described. Since the antibody to AST-CC was not available, we used ISH with a probe specific for a transcript encoding this neuropeptide to map its expression in the CNS of pharate 4–5th instar larvae (Fig. 3a, b). Neurons labelled with AST-CC probe were then identified with antibodies to bursicon, CCAP and MIP. This approach revealed a very strong AST-CC expression in cells 27/704 of the SG-TG1-3 (Fig. 3a) which also produce bursicon and ETHR-A (Fig. 2e, e’). In contrast, AG1-7 showed low AST-CC transcript levels in IN-704 that co-express CCAP, MIPs and ETHR-A (Figs. 2g–h’, 3b,b’).

Combination of ISH and IHC was also used to identify peptidergic content of VL8 and PM9 neurons expressing ETHR-A in the posterior TAG. Paired lateral VL8 neurons were labelled using ISH with sNPF probe followed by IHC with antibodies to MIP and RFamide (the latter antibody reacts with the C-terminal motif of sNPFs). This combined staining showed that VL8 are the only large lateral neurons coexpressing MIPs and sNPFs in the TAG (Fig. 3c, c’). To determine peripheral projections of VL8 neurons we then used whole larvae preparations for IHC with antibodies to MIP and RFamide. This technique revealed axonal processes of VL8 neurons that project via ventral nerves of the TAG (Fig. 3c’) and terminate on muscle surface in the posterolateral sides of the last 9th segment (Fig. 3d). Detected peripheral axons and especially axon terminals contain numerous varicosities indicating that neuropeptides sNPFs and MIPs are released from these putative neurohemal sites.

Since expression patterns of CCH1 has not been previously described, we used ISH with a CCH1 probe and IHC with a newly generated CCH1 antibody to map its spatial distribution. Both methods detected two prominent clusters of 4–6 neurons in the TAG (Fig. 3e, f) that resembled PM8 and PM9 neurons expressing CT and MIPs (Figs. 2h’, 3c’;^28^). Following IHC using antibodies to CCH1, CT and MIP confirmed colocalization of all three neuropeptides in these neurons (Fig. 3c’, f). As shown in this study, cluster of posterior PM9 also produce ETHR-A (Fig. 2h, h’). Parallel double staining of whole larvae preparations with antibodies to CCH1 and CT revealed that these medial neurons participate in elaborate innervation of the hindgut (Fig. 3g).

**Figure 4:**
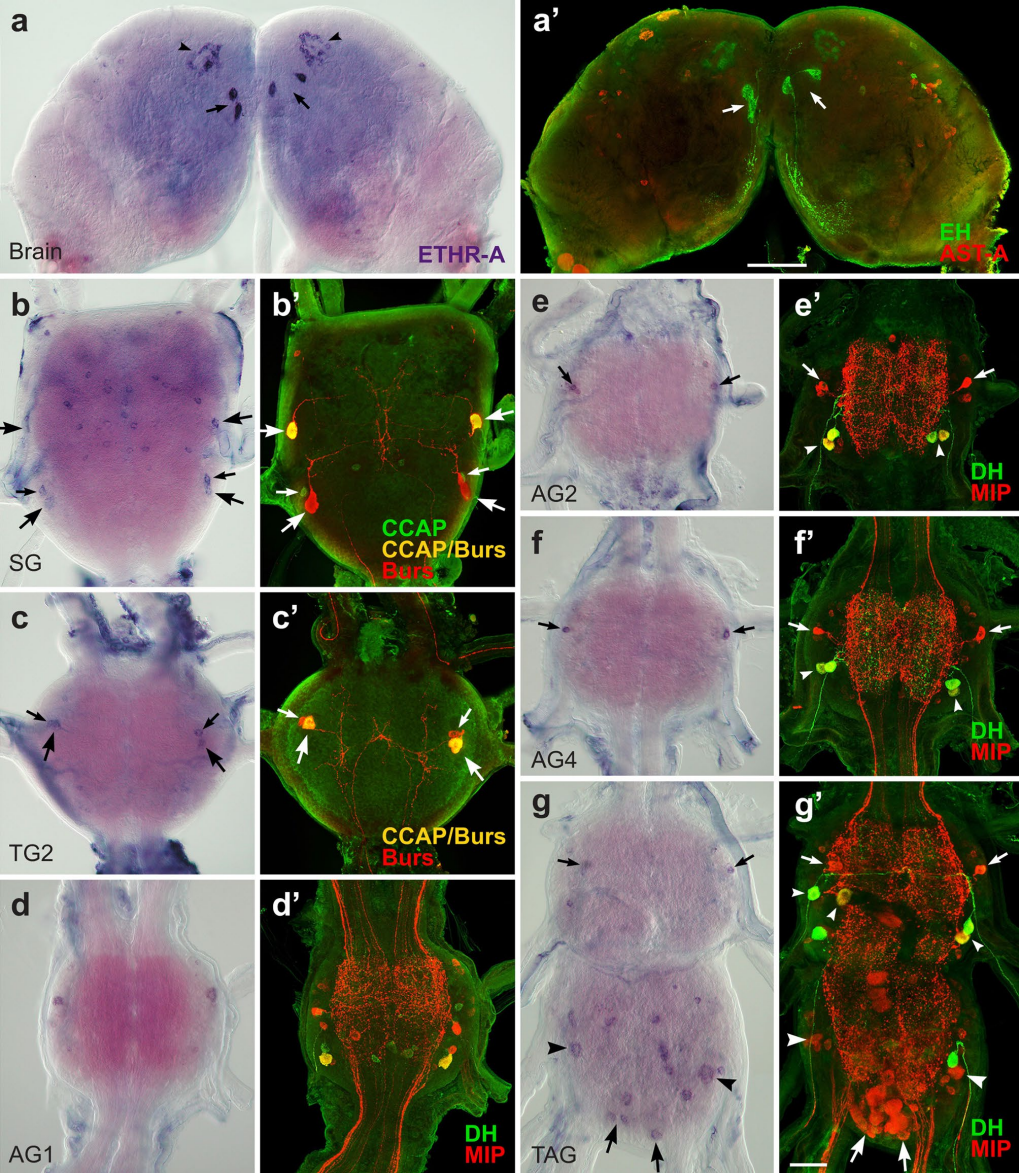
ETHR-A expression in the CNS of pharate pupae. (**a–g’**) Neurons detected by ISH with ETHR-A probe were identified by subsequent staining with various antibodies. (**a**, **a′**) ETHR-A transcript detected in EH-producing VM neurons (arrows; green) and in two clusters of 20–30 small neurons in the brain (arrowheads). (**b**, **b′**) ETHR-A expression in two pairs of NS-27 producing bursicon and CCAP (arrows; yellow) or bursicon only (arrows; red), posterior IN-704 containing CCAP (small arrows; green) and in ∼20 unidentified neurons of the SG. (**c**, **c’**) Colocalization of ETHR-A with bursicon and CCAP in NS-27 (large arrows; yellow) and bursicon only in IN-704 of the TG2 (small arrows; red). (**d**, **d’**) Unidentified pair of ETHR-A neurons in the AG1 failed to react with antibodies to DH (green) and MIP (red). (**e–g´**) ETHR-A expression in IN-704 stained with antibody to MIPs (arrows; red) in the AG2-7. Note the absence of ETHR-A in cells L_2,3_ producing kinins, DHs and MIPs (small arrowheads; green/yellow). (**g**, **g’**) Colocalization of ETHR-A and MIP in VL8 cells (arrowheads; red) and PM9 (large arrows; red) in the posterior TAG. ETHR-A was detected in additional 4–5 pairs of unidentified neurons. Scale bars a, a´ = 100 µm, b–g´ = 50 µm.

The initiation of metamorphosis in pharate pupae resulted in reconstruction of the CNS and numerous changes in ETHR-A expression when compared with pharate larvae. Only a few identified larval neurons retained ETHR-A production in pharate pupae that included VM cells in the brain (Fig. 4a, a’) and neurons NS-27, IN-704, VL8 and PM9 in the ventral ganglia (Fig. 4b–g’). Remaining ETHR-A neurons disappeared probably due to the programmed cell death or ETHR-A expression was lost in specific cells that survived metamorphosis. On the other hand, ETHR-A was detected in two novel clusters of 20–30 small neurons in each lateral protocerebrum and 5–6 small neurons scattered on the brain surface (Fig. 4a). The following neurons showed ETHR-A expression in the ventral ganglia—in the SG we detected anterior and posterior pair of cells NS-27 producing AST-CC, bursicon and CCAP, or AST-CC and bursicon, plus posterior pair of IN-704 containing AST-CC and CCAP, and a new group of ∼20–24 dorsal unidentified neurons (Fig. 4b, b’). NS-27 expressing AST-CC, bursicon and CCAP, plus IN-704 containing AST-CC and bursicon were found in the TG1-3 (Fig. 4c, c’). Unidentified pair of neurons was observed in the AG1 (Fig. 4d, d’), while each AG2-7 showed ETHR-A expression in a pair of IN-704 producing AST-CC, CCAP and MIPs and 2–3 small neurons (Fig. 4e–g’). ETHR-A also overlaps with sNPFs and MIPs in VL8 neurons and CT, MIPs and CCH1 in PM9 cells of the posterior TAG (Fig. 4g, g’). This ganglion also showed additional group of ∼10 new unidentified smaller cells (Fig. 4g). Interestingly, ETHR-A transcript was not detected in neurosecretory cells L_2,3_ which apparently survived metamorphosis and continued to produce kinins, DHs and MIPs (Fig. 4e’–g’). ETHR-A also disappeared from posterior DLT neurons producing NPF in the TG2-3 and anterior AST-A neurons in the AG1-8.

**Figure 5:**
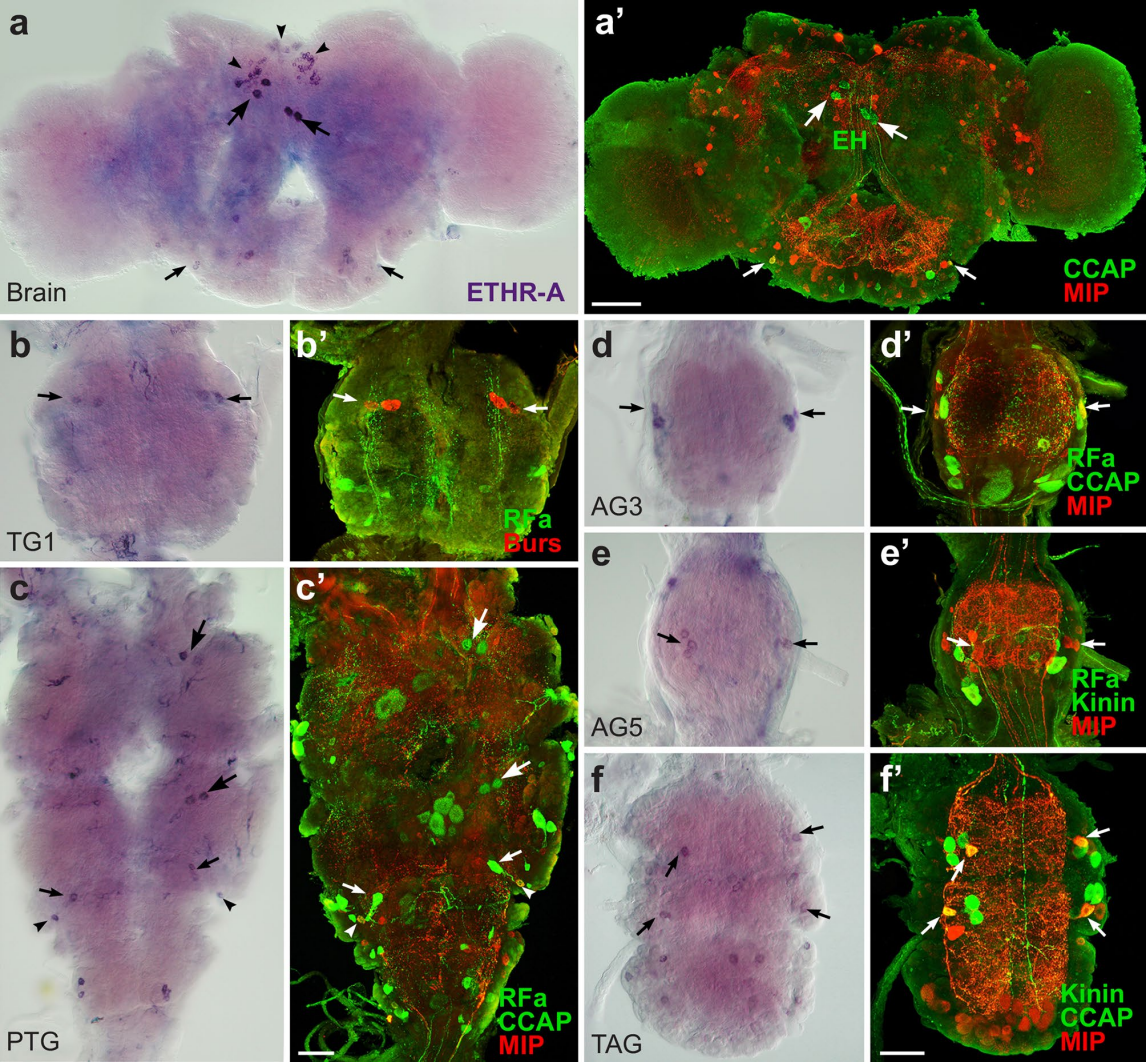
ETHR-A expression in the CNS of pharate adults. (**a–fʼ**) Neurons identified by ISH with ETHR-A probe and subsequent staining of the same ganglia with various antibodies. (**a**, **aʼ**) ETHR-A transcript in two pairs of VM cells producing EH (large arrows; green) and three clusters of small dorsomedial neurons in the brain (arrowheads). A pair of IN-704 producing ETHR-A in the SG stained with antibodies to CCAP and MIPs (small arrows; yellow). (**b**, **bʼ**) NS-27 and IN-704 showing coexpression of ETHR-A and bursicon in the TG1 (arrows; red) and (**c**, **cʼ**) the same neurons producing ETHR-A and CCAP in the fused TG2,3 (large arrows; green). (**c**, **cʼ**) Colocalization of ETHR-A with CCAP in NS-27 (small arrows; green) and ETHR-A with CCAP and MIP in IN-704 (arrowheads; yellow) of the fused AG1,2. (**d–fʼ**) ETHR-A expression in IN-704 producing CCAP and MIPs in the AG3-5 and TAG (arrows; red/yellow). (**f**) Note ETHR-A signal in additional neurons of the posterior TAG. Scale bars a,a´ = 100 µm, b-f´ = 50 µm.

**Figure 6:**
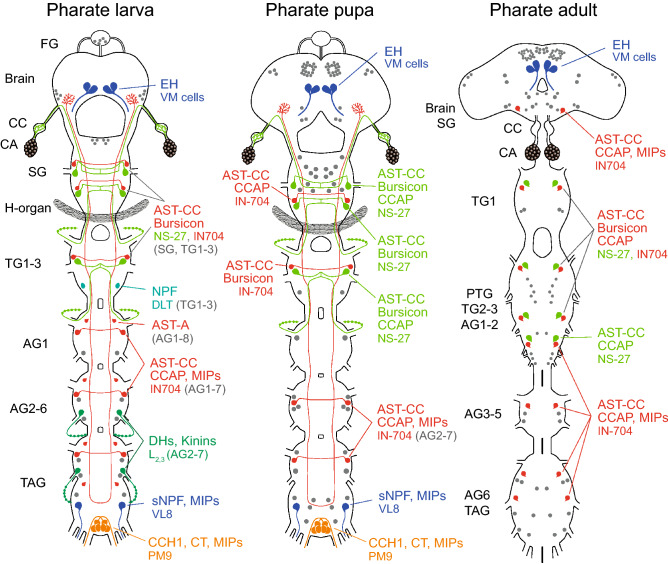
Schematic drawings depicting changes of ETHR-A expression in the CNS, CA and H-organ of pharate larvae, pupae and adults. Most larval ETHR-A neurons produce a large array of neuropeptides. However, number of peptidergic neurons decreased during metamorphosis and newly differentiated neurons in pharate pupae and adults do not seem to produce any known neuropeptide(s). Each identified type of peptidergic neuron is labelled by different color, while remaining unidentified ETHR-A neurons are labelled grey. Note very strong and consistent expression of ETHR-A in the CA and H-organ. The latter organ disappears after pupation.

Adult development during pupal stage is associated with dramatic reorganization of the CNS—the brain develops optic and antennal lobes and fuses with SG; thoracic ganglia TG2,3 and abdominal ganglia AG1,2 form a large pterothoracic ganglion (PTG) and AG6-8 fuse into the TAG. This results in further changes and reduction in ETHR-A expression in pharate adults. At this stage we were able to identify only three types of neurons producing ETHR-A—VM cells in the brain, plus NS-27 and IN-704 in the ventral nerve cord (Fig. 5a–f’). Similarly as described in pharate pupae, the strongest ETHR-A expression was detected in VM cells and two clusters of 30–40 lateral neurons in the brain (Fig. 5a, a’). A new cluster of 20–30 small neurons appeared in the dorsomedial protocerebrum (Fig. 5a) and a group of ∼10–12 small neurons was observed in the dorsal optic lobes in some preparations. Consistent ETHR-A staining was confirmed in NS-27 and/or IN-704, but these neurons produced a different mixture of neuropeptides in each ganglion. In the SG ETHR-A transcript was found in a pair of IN-704 containing AST-CC, CCAP and MIPs and about 10–12 unidentified neurons. ETHR-A expression in the TG1 and fused TG2,3 was restricted to cells 27/704 producing AST-CC, bursicon and CCAP (Fig. 5b–c’). In the fused AG1,2 NS-27 coexpressed ETHR-A with AST-CC and CCAP, while in IN-704 ETHR-A overlapped with AST-CC, CCAP and MIPs (Fig. 5). Additional 2–3 unidentified lateral neurons were found in the posterolateral part of the AG2 (Fig. 5c). In the unfused AG3-5 and TAG, ETHR-A transcript was only detected in IN-704 containing AST-CC, CCAP and MIPs and adjacent unidentified larger neurons (Fig. 5d–f’). In the posterior TAG we observed additional 3–5 pairs of neurons (Fig. 5f). Expression of ETHR-A in pharate larvae, pupae and adults and its colocalization with known neuropeptides is depicted in schematic drawings in Fig. 6.

### ETHR-B expression in the CNS

**Figure 7:**
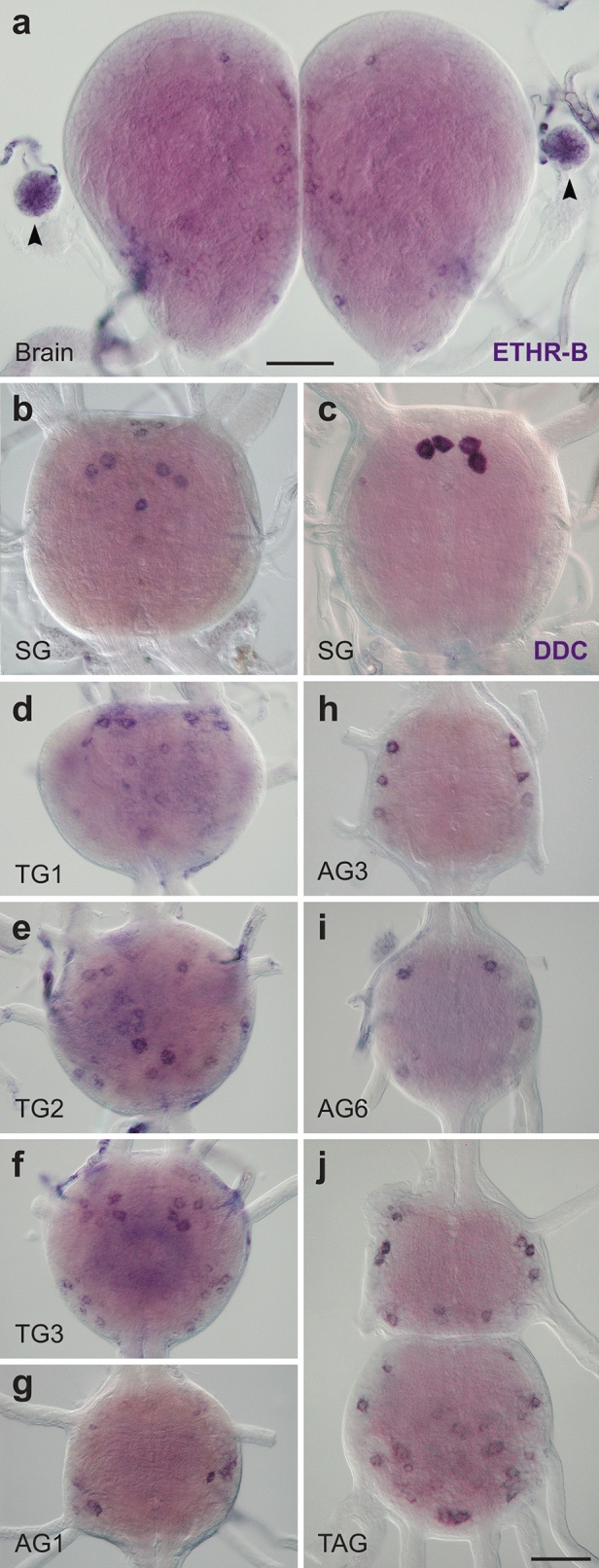
ETHR-B expression in the CNS of pharate larvae. (**a-j**) Neurons stained by ISH with probes for ETHR-B (**a–b**, **d–j**) or DDC (**c**). (**a**) ETHR-B transcript detected in numerous small medial neurons of the brain and endocrine cells of the CA (arrowheads). (**b**) In the SG ETHR-B was observed in several small medial neurons and four large cells that resemble those expressing DDC in the same ganglion (**c**). (**d–f**) ETHR-B expression in 4–8 large anterior cells and 6–10 additional neurons in the TG1-3. (**g–j**) ETHR-B staining in 4–6 pairs of dorsolateral neurons in the AG1-7 and 10–14 paired neurons in the posterior TAG. Scale bars = 50 μm.

ETHR-B was expressed in a large number of neurons which increased during metamorphosis, but only a few of them were identified. In pharate larvae, about 10–15 pairs of neurons were observed on the dorsal surface of the brain, 6–10 small neurons in the FG and strong expression was detected in the CA (Fig. 7a). In the SG ETHR-B transcript was localized in several small medial neurons and four large cells that resemble those producing DOPA dexarboxylase (DDC; Fig. 7b–c). A group of 4–8 large ETHR-B neurons and additional 6–10 paired neurons were stained in each thoracic ganglion (Fig. 7d–f). The AG1-7 showed consistent ETHR-B expression in 4–6 pairs of dorso-lateral neurons of different staining intensity (Fig. 7g–j). Interestingly, small lateral ETHR-B neurons in the AG1 (Fig. 7g) were apparently distinct from larger cells detected in the AG2-7 (Fig. 7h–j). Likewise, the expression pattern of ∼10–14 pairs of neurons stained in the AG8 was completely different from the remaining ganglia (Fig. 7j).

**Figure 8:**
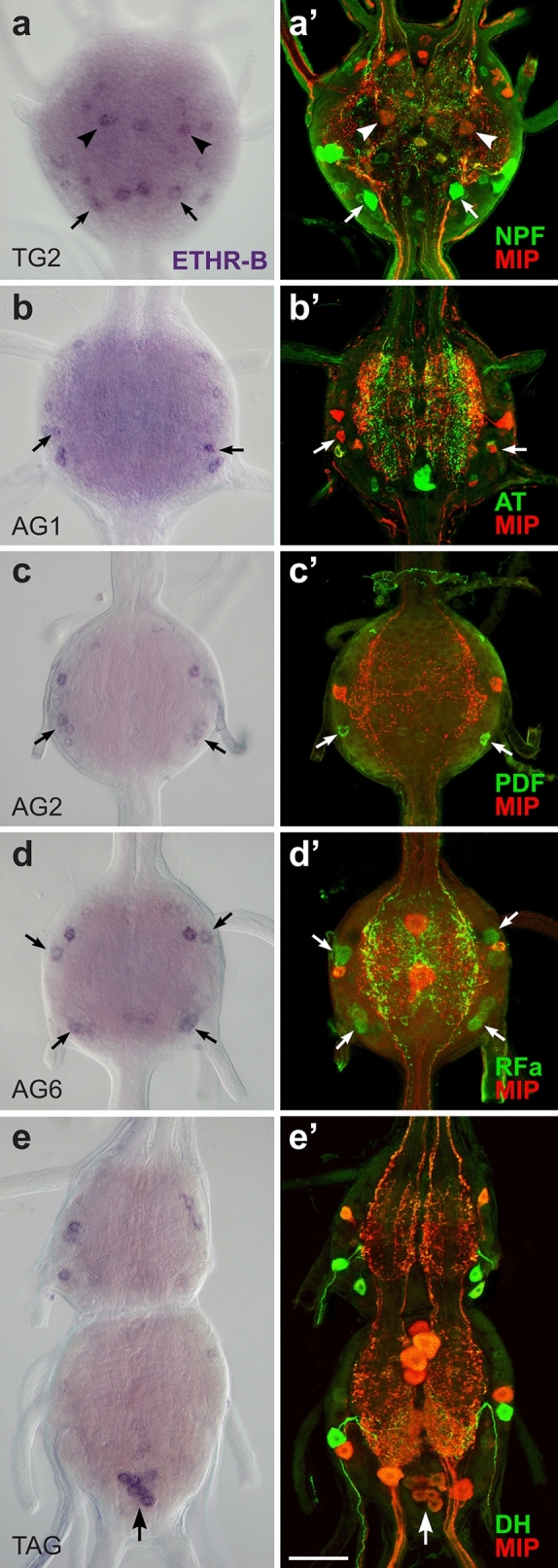
Identification of ETHR-B neurons in the CNS of pharate larvae. (**a**–**e**ʼ) ISH with ETHR-B probe and following staining of the same ganglia with various antibodies. (**a**, **a**’) Coexpression of ETHR-B with MIP (arrowheads; red) and NPF (arrows; green) in the TG2. (**b**, **b**’) Colocalization of ETHR-B transcript and MIP (arrows; red) in a pair of small neurons of the AG1. (**c**, **c**’) A posterolateral pair of ETHR-B neurons stained with PDF antibody in the AG2 (arrows; green). (**d**, **d**’) Coexpression of ETHR-B and RFamide-like peptide (arrows; green) in 2–3 pairs of lateral neurons in the AG6. (**e**, **e**’) A cluster of PM9 neurons expressing ETHR-B in the posterior TAG identified with MIP antibody (arrow; red). Note that other ETHR-B neurons failed to react with antibodies to MIP (red; **a**’–**e**’), AT (green; **b**’) or DH (green; e´). Scale bar = 50 μm.

ISH combined with double or triple staining with neuropeptide antibodies revealed that only a few ETHR-B neurons are peptidergic. These include a pair of anterior neurons in the TG1-3 producing MIPs and a posterior pair of DLT cells expressing NPF (Fig. 8a, a’). MIP was also detected in a pair of small lateral neurons in the AG1 (Fig. 8b, b´), while pigment dispersing factor (PDF)-like and RFamide-like peptides were observed in lateral cells of the AG2-7 (Fig. 8c–d’). In the posterior TAG ETHR-B transcript was identified in a cluster of PM9 neurons that produce CT, MIPs and CCH1 (Fig. 8e, e’). Notably, DLT and PM9 are the only neurons coexpressing both ETHR subtypes.

**Figure 9:**
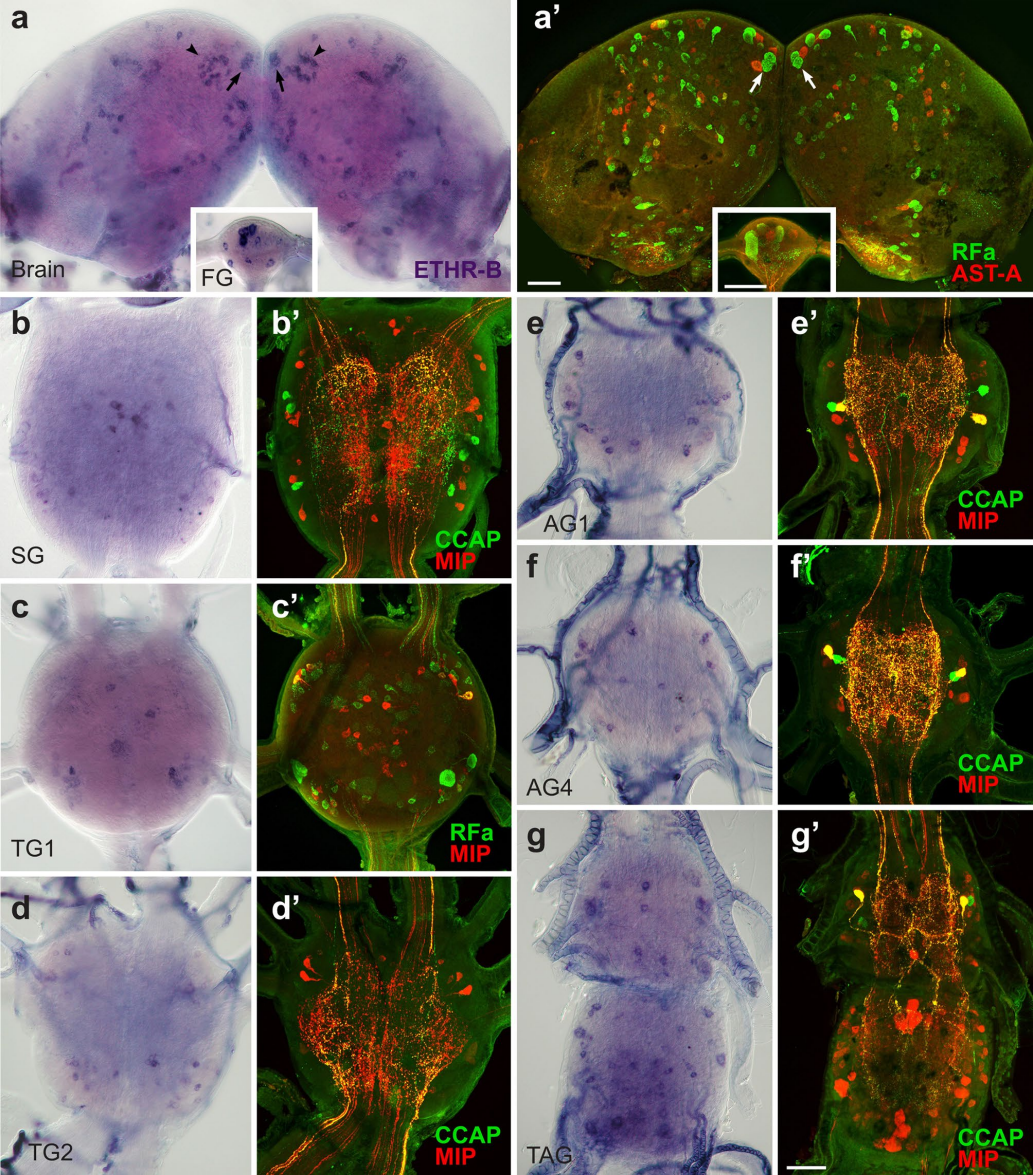
ETHR-B expression in the CNS of pharate pupae. (**a–gʼ**) ISH using ETHR-B probe followed by immunostaining of the same ganglia with antibodies to various neuropeptides. (**a–a'**) Brain cells IIa_5_ producing ETHR-B and MS (RFa antibody binds to MS C-terminal) (arrows; green) and two clusters of small neurons that probably coexpress both receptor isoforms (arrowheads). Other small ETHR-B neurons were not labelled with antibodies to RFamide (green) and AST-A (red). (**a**, **a’**; inset) ETHR-B staining in 8–10 small neurons of the FG that was not colocalized with RFamide (green) or AST-A (red). (**b–g’**) Numerous ETHR-B neurons in ventral ganglia that failed to react with antibodies to CCAP and MIP (**bʼ**, **dʼ–gʼ**; green/red) or RFamide and MIP (**cʼ**; green/red). Scale bars = 50 μm.

**Figure 10:**
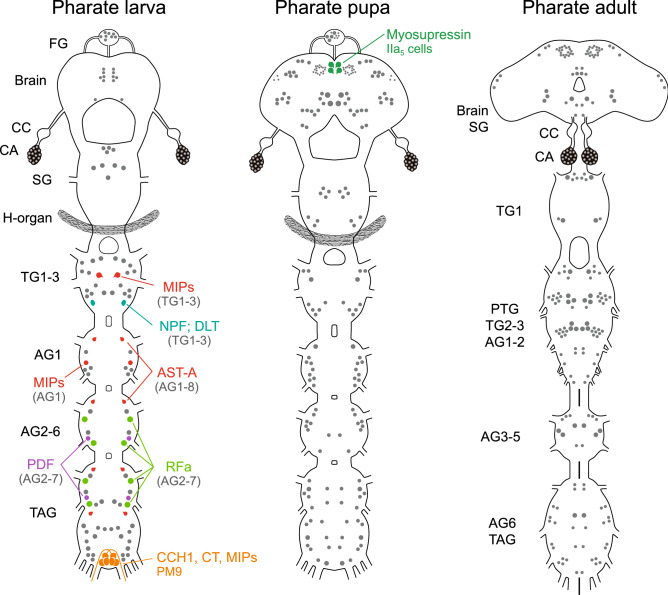
Schematic drawings showing expression of ETHR-B in the CNS, CA and H-organ of pharate larvae, pupae and adults. Only a few neurons in the ventral nerve cord of pharate larvae and in the brain of pharate pupae produce neuropeptides. Each type of these peptidergic neuron is labelled with a different color. Note considerable changes of expression pattern and increasing number of ETHR-B neurons during metamorphosis. Most of these unidentified neurons may be aminergic. Strong ETHR-B expression was also detected in the CA and H-organ.

Expression pattern of ETHR-B considerably changed in the CNS of pharate pupae (Fig. 9). In the brain, we observed an increased number of ∼120 ETHR-B neurons that included four neurosecretory cells IIa_5_ producing myosupressin (MS) (Fig. 9a, a´) and paired clusters of 20–30 small neurons that are probably identical to those expressing ETHR-A (Figs. 4a, 9a). Remaining small neurons scattered all over the brain and FG were not identified (Fig. 9a, a’; inset). ETHR-B transcript was found in many neurons of the ventral nerve cord, which do not seem to produce any known neuropeptide (Fig. 9b–g’). In pharate adults ETHR-B was also detected in paired clusters of 20–30 small neurons in the protocerebrum, plus numerous cells in the brain and ventral nerve cord, but none of them overlapped with peptidergic cells stained with our antibodies (see Supplementary Fig. S1 and S2 online). Expression of ETHR-B in pharate larvae, pupae and adults and its colocalization with known neuropeptides is shown in schematic drawings (Fig. 10).

### Roles of different neuropeptides during the ecdysis sequence

Expression of ETHRs in peptidergic neurons suggests that PETH and ETH action on these specific targets induces the release of multiple neuropeptides to control specific phases of the ecdysis sequence. Indeed, previous experiments showed that kinins and DHs control pre-ecdysis, while a cascade of neuropeptides EH, CCAP, MIPs and bursicon control ecdysis and post-ecdysis^7,19^. However, ETHR neurons produce a large array of other neuropeptides (e.g. AST-CC, MS, PDF, RFamides, NPF, sNPFs, MIPs, CCH--CT-MIPs) that may activate or suppress different phases of the ecdysis sequence but their roles have not been elucidated. Therefore, we used electrophysiology technique to monitor neuropeptide-induced neuronal motor burst patterns that corresponded to distinct pre-ecdysis behaviors, or initiation and termination of ecdysis.

### Neuropeptides controlling pre-ecdysis I and II

**Figure 11:**
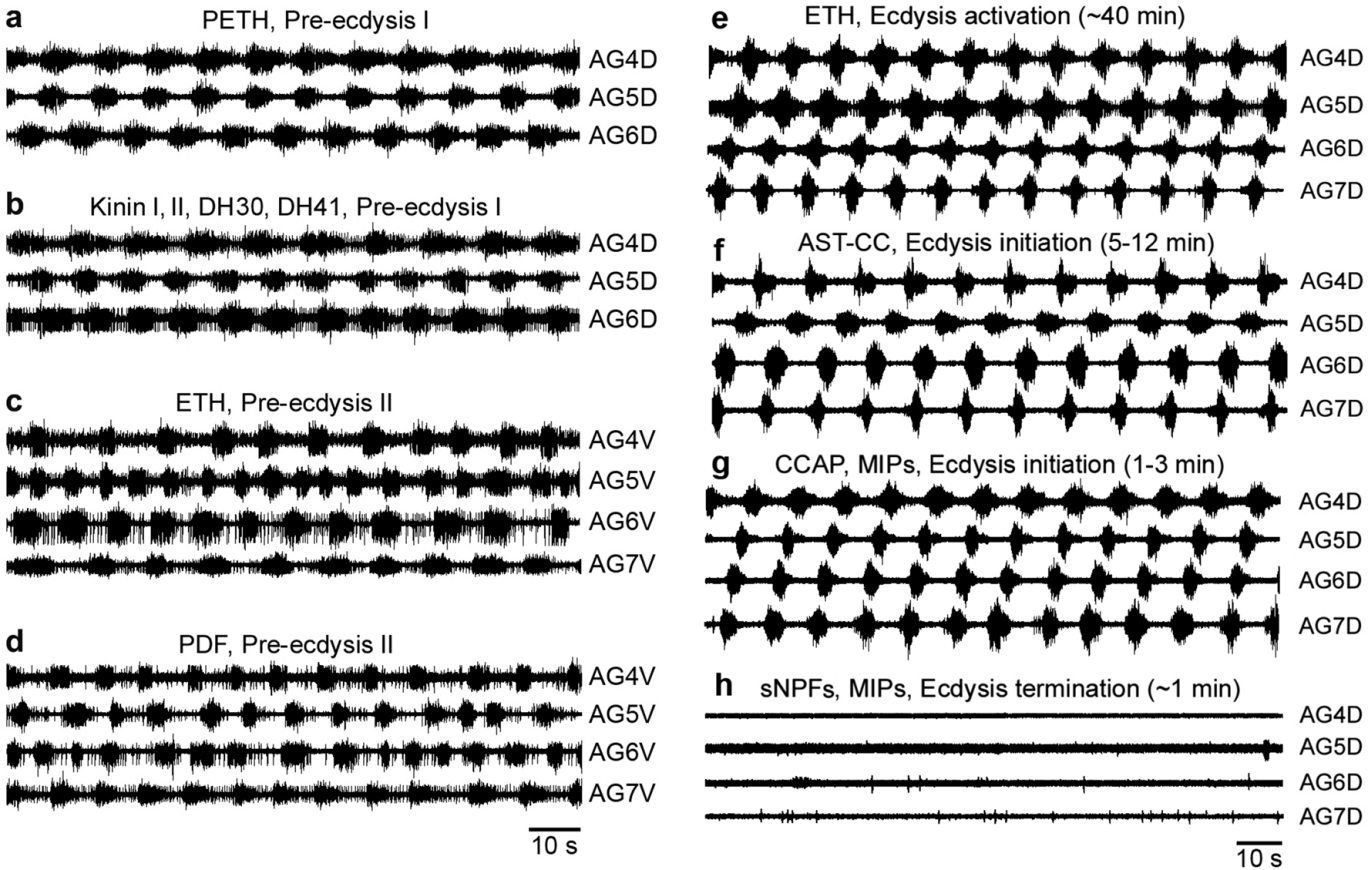
Effects of different peptides on the initiation or termination of pre-ecdysis or ecdysis bursts in the isolated CNS of pharate 5th instar larvae. (**a**) PETH-induced bursts characteristic for pre-ecdysis I recorded in dorsal nerves of the abdominal ganglia 4–6 (AG4-6D). (**b**) Very similar burst patterns were recorded in the desheathed AG4-6D after application of a mixture of kinins and DHs. (**c**) ETH-induced pre-ecdysis II bursts in ventral nerves of AG4-7 (AG4-7 V) that closely correspond to those evoked by PDF (**d**). (**e**) The isolated CNS of pharate larvae treated with ETH showed pre-ecdysis for ∼40 min and then switched to ecdysis motor patterns that were recorded in dorsal nerves of abdominal ganglia 4–7 (AG4-7D). (**f**) Application of AST-CC on the desheathed CNS evoked characteristic ecdysis bursts in 5–12 min without activation of pre-ecdysis motor program. (**g**) A mixture of CCAP and MIPs also induced ecdysis bursts in 1–3 min. (**h**) ETH-induced ecdysis activity were irreversibly terminated in ∼1 min after exposure of the desheathed CNS to a mixture of sNPFs and MIPs.

Based on previous reports^7^ we hypothesized that in *B. mori* activation of neurons L_3,4_ by PETH or ETH elicits pre-ecdysis I through the release of kinins and DHs. As expected, application of a mixture containing kinins I, II, and DH30, 41 (0.3–1 μM each) to desheathed CNS (n = 7) induced within 3–5 min strong bursts in dorsal nerves that lasted for ~ 30–40 min and were indistinguishable from PETH-induced pre-ecdysis I bursts (Fig. 11a, b). Individually applied kinins or DHs (1 μM) induced noisier pre-ecdysis I burst patterns (n = 6) (data not shown). Washout of kinins and DHs abolished pre-ecdysis I bursts, while repeated application of these peptides restored pre-ecdysis I motor patterns (n = 7).

Previous studies in pharate larvae or pupae of *B. mori* and *M. sexta* showed that each abdominal ganglion contains the entire circuitry for pre-ecdysis II^30,31^. In search for a proximal signal controlling pre-ecdysis II we therefore tested neuropeptides produced by each abdominal ganglion. The best candidates were segmental abdominal ETHR-B neurons producing PDF- and RFamide-like peptides (Fig. 8c–d´). Indeed, our in vitro experiments indicate that PDF may be a proximal signal for initiation of pre-ecdysis II. Exposure of the desheathed CNS to PDF (0.3–1 μM) induced in 3–5 min asynchronous motoneuron activity in ventral nerves of AG2-7 closely corresponding to ETH-evoked pre-ecdysis II bursts in these ganglia (Fig. 11c, d). PDF washout led to cessation of pre-ecdysis II, but these bursts were restored following peptide reapplication (n = 8).

To compare effects of kinins and DHs or PDF with those evoked by PETH or ETH we treated the isolated nerve cords with the latter peptides (0.3–1 μM) and monitored pre-ecdysis activity in dorsal and ventral nerves (Fig. 11a, c). In 5–7 min all isolated nerve cords responded to PETH or ETH by burst patterns characteristic for pre-ecdysis I and II and continued to show these bursts even after peptide washout (n = 14). These data further confirm that PETH and ETH act via downstream signaling pathways to control pre-ecdysis behaviors that probably include kinins, DHs and PDF. On the other hand, a mixture of RFamides I, II, III (0.3–1 μM each) which were hypothesized to participate in pre-ecdysis, failed to induce any patterned bursts in the desheathed CNS (n = 7) (data not shown) and their role in the ecdysis sequence remains enigmatic.

### Neuropeptides controlling ecdysis initiation

Experimental evidence clearly showed that a network of cells 27/704 is crucial for regulation of insect ecdysis behavior^4,7,8,30,32^. Strong AST-CC expression in cells 27/704 of the SG-TG1-3 (Fig. 3a) therefore indicated a possible role of this neuropeptide in the ecdysis sequence. Indeed, exposure of the desheathed CNS to AST-CC (0,3–1 μM) led to characteristic ecdysis bursts in 5–12 min that were very similar to those induced by ETH (Fig. 11e, f). Interestingly, AST-CC induced this motor pattern only in the CNS dissected 2–6 h prior to ecdysis (n = 14), while earlier pharate or feeding stages were irresponsive to this peptide (n = 9) (compare with CCAP/MIPs effects below). These data indicate that AST-CC receptor is expressed or activated only several hours prior to the initiation of the ecdysis sequence.

CCAP and MIPs produced by ETHR-A interneurons IN-704 act as proximal activators of the ecdysis behavior in *M. sexta* and *D. melanogaster*^7,8,32,33^. To determine if these neuropeptides control ecdysis in *B. mori*, we applied a mixture of CCAP (1 μM) and MIP-I-VII (3 μM total) to the isolated desheathed CNS (n = 8). As expected, the mixture induced clear and characteristic ecdysis bursts within 1–3 min in all ganglia AG1-7 (Fig. 11g). In contrast to AST-CC, neuropeptides CCAP and MIPs activated normal ecdysis motor program in the desheathed CNS of early pharate or feeding 5th instar larvae (n = 7; Fig. S3). Washout of CCAP and MIPs waned the ecdysis activity, whereas repeated applications of the mixture on the same CNS restored normal ecdysis bursts. CCAP alone was less effective and induced clear ecdysis patterns only in the anterior ganglia AG1-3, whereas posterior ganglia AG5-7 showed either noisy bursts or no discernable rhythmic activity (n = 4). Application of MIP-I only or a mixture of MIP-I-VII induced weak prolonged synchronous bursts or no rhythmic pattern (n = 5; data not shown).

### Neuropeptides controlling ecdysis termination

Under normal conditions, the ecdysis behavior lasts for ∼10 min and terminates immediately after the old cuticle is completely shed from the last abdominal segment. However, pharate larvae injected with ETH at 8–15 h prior to ecdysis fail to shed the old cuticle and show strong ecdysis contractions for 1–2 h. This indicates that a sensory input probably located in abdominal segments is required for ecdysis termination. To determine a site of sensory input that terminates the ecdysis behavior, we peeled off a narrow ring of the old cuticle on abdominal segments 1, 3, 5 or 6 of late pharate 5th instar larvae 1–3 h prior to ecdysis. These larvae invariably initiated normal pre-ecdysis behaviors at the expected time and after ∼1 h switched to peristaltic waves of ecdysis contractions. This resulted in shedding of abdominal segments posterior of the peeled ring, while the anterior segments remained covered with the old cuticle. In spite of presence of the old cuticle on the head, thorax and anterior abdomen, strong ecdysis contractions ceased immediately after the posterior cuticle was shed from the last abdominal segment in all larvae (n = 14). These experiments indicated that the sensory input for ecdysis termination is located in the last 9th abdominal segment.

As described above, projections of lateral VL8 neurons coexpressing ETHR-A and neuropeptides sNPFs and MIPs terminate on the muscle surface in the 9th abdominal segment (Fig. 2h, h’, 3c–d) which suggests a possible role of these inhibitory neuropeptides in ecdysis termination. To test this hypothesis, we first activated desheathed CNS by ETH and after initiation of clear ecdysis bursts in 27–35 min, we added a mixture of sNPF-I-III (1 μM total) and MIP-I-VII (3 μM total) into the bath. This treatment invariably abolished bursting activity within 30–60 s and all monitored ganglia (AG1-7) remained inactive for 5–20 min of the recording session (n = 10) (Fig. 11h). The inhibitory effect of sNPF-I-III and MIP-I-VII was irreversible and repeated washout of these peptides never restored ETH-induced ecdysis bursts.

### Quantification of ETHRs levels in the CNS and peripheral organs

**Figure 12:**
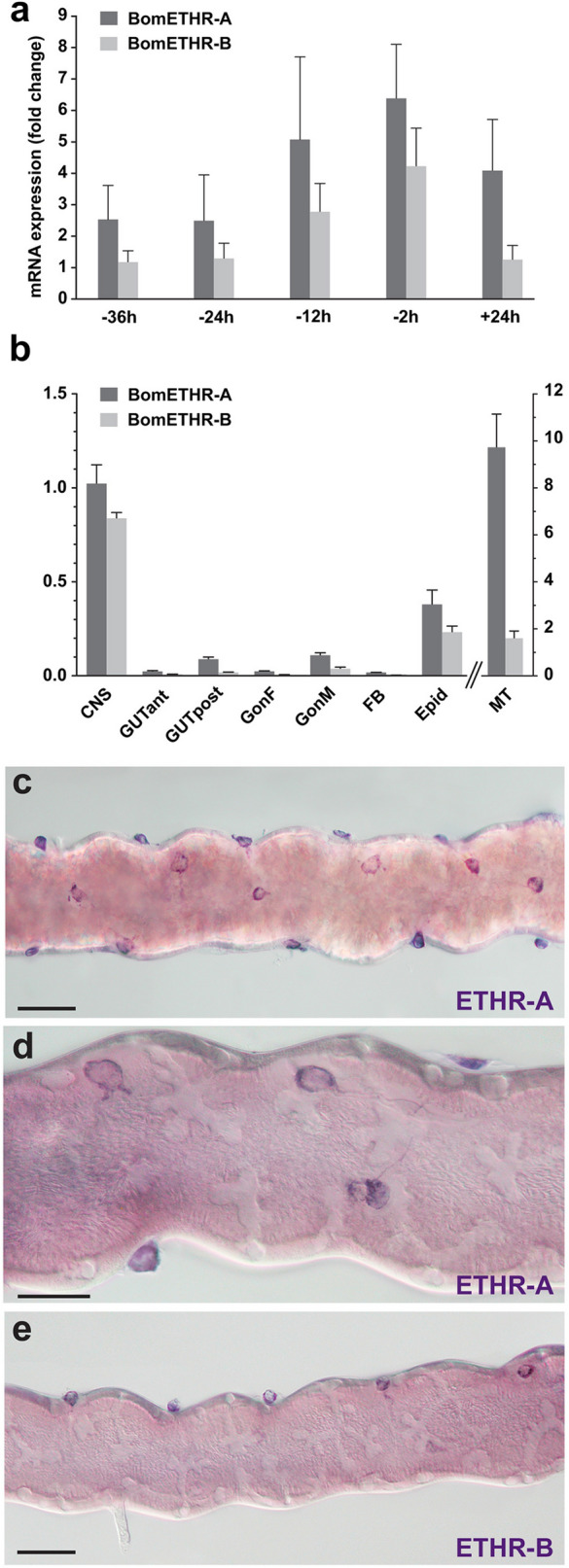
Temporal changes and spatial distribution of ETHRs expression. (**a**) Relative transcripts levels of ETHR-A and ETHR-B in the CNS measured at different time points in pharate or ecdysed 5th instar larvae. 0 h represents ecdysis onset. RNA used for RT-qPCR was extracted from CNS with attached CA and H-organ. (**b**) Expression levels of ETHR-A and ETHR-B in different tissues of pharate 5th instar larvae. Error bars indicate standard error of the mean (n = 3). CNS (central nervous system), GUTant (anterior gut), GUTpost (posterior gut), GonF (female gonads) GonM (male gonads), FB (fat body), Epid (epidermis), MT (Malpighian tubules). (**c–e**) ETHR-A and ETHR-B expression in MT cells attached to surface of the Malpighian tubules. Scale bars c, e = 50 µm, d = 25 µm.

Expression levels of ETHR-A and ETHR-B in the CNS at different time points before and after larval ecdysis were determined using RT-qPCR (Fig. 12a). Increased levels of both receptor subtypes were detected in the CNS ~ 12–15 h prior to ecdysis and reached a peak expression at the brown mandible stage (− 2–4 h). Notably, feeding 5th instar larvae 24 h after ecdysis still showed detectable levels of ETHR transcripts.

Since the highest levels of both transcripts in the CNS were detected at − 2–4 h, we used this stage to analyze ETHRs in other peripheral organs. The strongest expression was found in the Malpighian tubules, while moderate-weak increase was observed in the epidermis, male gonads and posterior gut. No obvious ETHRs levels were detected in the fat body, ovaries, or anterior gut (Fig. 12b).

ISH was used to identify cellular source(s) of ETHR-A and ETHR-B expression in the Malpighian tubules of pharate 5th instar larvae dissected at different stages. Apparent expression of both receptor subtypes was detected in relatively small rounded cells with short cytoplasmic processes (10–15 µm in diameter) that were individually attached to surface of the Malpighian tubules (Fig. 12c–e). Since they have not been previously described, we designated them as MT cells. Expression of both ETHRs in these cells was restricted to the brown mandible stage (− 2–4 h), whereas no staining was detected in earlier and later pharate stages or freshly ecdysed 5th instar larvae. High levels of ETHRs in the MT cells indicates possible involvement of ETH signaling in regulation of water balance. These cells could be fibroblasts or myoblasts, but their specific function needs further examination. We also used ISH for localization of ETHRs in the gonads, gut, and epidermis with attached Verson´s glands and small gland cells at the base of bristles, but the results were inconclusive due to high background and/or unspecific staining of tissues containing alkaline phosphatase.

## Discussion

### Organization and expression of ETHRs

ETHs are produced by endocrine Inka cells of diverse representatives of hemimetabolous and holometabolous insects. They act as command factors to activate downstream regulatory pathways within the CNS controlling a behavioral sequence critical for successful shedding of the old cuticle^1,4,10,21^. Identification and localization of ETH receptors within the CNS and peripheral tissues is a key step to pinpoint downstream factors and elucidate their functions during the ecdysis sequence and associated physiological processes. The *ethr* gene encoding two alternatively spliced G protein-coupled receptors (ETHR-A and ETHR-B) was first identified in *D. melanogaster*^16,34^. Following studies showed that alternative splicing of primary *ethr* transcript is highly conserved in representatives of diverse arthropod species and conducted phylogenetic analyses segregated ETHR-A and ETHR-B into separate clades^7,8,11,13,35^. In *B. mori*, ETHR-A and ETHR-B differ in sequences at the C-termini encoded by two alternatively spliced 3′-exons of a single *ethr* gene (Fig. 10a).

Using in vitro assay with CHO cells, we observed obvious differences in the affinity of each receptor isoform to PETH or ETH. Although the concentration–response curves showed similar sensitivity of ETHR-B to both peptides, ETHR-A was ~ 20-fold less sensitive to PETH than to ETH (Fig. 10b, c). Different affinities of two ETHR subtypes to ETHs were also observed in other insects^7,13,16,34,35^. These findings support the proposed idea of sequential activation of direct neuronal targets after ETH release from Inka cells. Distinct sensitivity of ETHR-A and ETHR-B together with their differential spatial distribution and expression levels in non-overlapping subsets of neurons^7,8^ suggest specific roles of each isoform in proper activation and orchestration of the ecdysis sequence. This hypothesis is supported by experiments in *D. melanogaster* showing that genetically altered levels of ETHR expression or manipulation of signaling pathways in specific neuronal ensembles influenced timing and duration of consecutive behavioral steps during the ecdysis sequence^19^. Therefore, distinct density of ETHR-A or ETHR-B in specific subsets of neurons, combined with differential sensitivity of these receptors, may underlie mechanisms controlling proper timing of responses to the same ligand. Sequential release of a large array of neuropeptides and other regulatory molecules controls strictly determined activation or termination of individual behavioral steps.

Mapping of ETHR-A and ETHR-B expression in *M. sexta* and *D. melanogaster* revealed mutually exclusive distribution of transcripts in discrete populations of neurons throughout the CNS. Almost all ETHR-A and only a few ETHR-B neurons are peptidergic, while identity of regulatory molecules produced by most ETHR-B neurons is unknown^7–9^. Careful examination of ETHRs expression in pharate larvae, pupae and adults of *B. mori* using ISH with probes specific for ETHR-A or ETHR-B followed by IHC with neuropeptide antibodies resulted in detection and identification of numerous neurons in the CNS. Each splice variant is differentially expressed in non-overlapping populations of central neurons, with a few exceptions where both receptor variants were colocalized in the same cells (Figs. 2, 8). Virtually all ETHR-A neurons in pharate larvae could be subdivided into separate subsets of neurons that are characterized by specific anatomy and production of a wide range of neuropeptides. These include EH in the brain VM cells, AST-CC, bursicon, CCAP and MIPs in cells 27/704 of the ventral nerve cord, kinins-DHs in L_2,3_ cells of AG2-7, and sNPFs with MIPs in VL8 neurons of the TAG. The only identified larval neurons coexpressing both ETHR subtypes are DLT cells producing NPF in thoracic ganglia and a cluster of PM8 neurons containing CCH1, CT and MIPs in the TAG. A few additional ETHR-B neurons express MS in the brain, MIPs in the TG1-3 and AG1, plus RFa- and PDF-like peptides in the AG2-7. However, a large group of various ETHR-B neurons is not peptidergic. Interestingly, most of these cells disappeared or lost expression of ETHR-A and ETHR-B during metamorphosis, while newly differentiated receptor neurons do not seem to produce any known neuropeptides and their modulators or transmitters remain to be identified. Thus signaling molecules of most ETHR neurons have not been characterized. Recent study in *D. melanogaster* revealed that some ETHR neurons produce acetylcholine, glutamate and GABA^9^, indicating that biogenic amines could be the main regulatory molecules in some of these uncharacterized neurons in *B. mori.* We detected DDC transcript (an enzyme converting DOPA to dopamine) in four large cells that resemble those expressing ETHR-B in the SG (Fig. 7b, c). However, colocalization of DDC and ETHR-B in these neurons needs to be confirmed.

This study revealed considerable changes in expression of ETHR-A and ETHR-B in the CNS of *B. mori* during development and metamorphosis that may reflect adaptations to different behavioral patterns and functions of ETH signaling in pharate larvae, pupae and adults. Studies in various insect models support this hypothesis. In *D. melanogaster* ETHR-A is required for successful shedding of the cuticle throughout the whole development, while ETHR-B may not be necessary for larval ecdysis, but is essential for pupal and adult ecdyses^9^. RNAi silencing of ETHR-A in *B. dorsalis* caused larval ecdysis failure, while no defects or phenotype was observed following ETHR-B knockdown^35^. On the contrary, ETH and ETHR-B play an essential role in reproduction of the female adults via regulation of JH production and vitellogenesis^24^. The global RNAi knockdown of ETHR-A in *T. castaneum* resulted in the failure of adult ecdysis, but the role of ETHR-B is less clear^12^. These data provide clues for elucidation of stage-specific roles of each receptor isoform. Moreover, developmental changes in ETHRs expression may reflect differences in behavioral patterns during larval, pupal and adult ecdyses described in other insects.

Previous studies in *M. sexta* have demonstrated that rising ecdysteroid levels induce expression of both, ETH and its precursor forms in Inka cells and ETHRs in the CNS^6,36^. Likewise, timing of ETHR expression and behavioral competence coincided with increased ecdysteroids levels in the mosquito *Aedes aegypti*^13^. Results of our RT-qPCR analysis indicate that changing transcript levels of ETHR-A and ETHR-B in the CNS of *Bombyx* pharate 5th instar larvae also correlate with increased ecdysteroid titers^37,38^. These data further confirm that ecdysteroid-induced expression of ETHRs is most likely mechanisms underlying sensitivity of central neurons to PETH and ETH.

### Roles of different neuropeptides in ETHR neurons of the CNS

The peptidergic nature of ETHR-A and several ETHR-B neurons suggests that PETH and ETH activate pre-ecdysis, ecdysis and post-ecdysis behaviors through the release of multiple neuropeptides within the CNS. Upon activation of these neurons a mixture of different excitatory and inhibitory neuropeptides is sequentially released to initiate a precisely coordinated set of motor programs essential for successful transition to the next developmental stage^1,4^. Electrophysiology experiments using isolated and desheated CNS indicated a specific role of abdominal ETHR-A neurons L_3,4_ producing kinins and DHs in pre-ecdysis I, while ETH action on EH cells and neurons 27/704 producing CCAP, MIPs and bursicon leads to initiation of the ecdysis and post-ecdysis behaviors^7,14,32,39^. Parallel molecular and genetic approaches in *D. melanogaster* revealed kinin as a downstream regulator of pre-ecdysis^8,19^ and similarly as described in moths, EH, CCAP, MIP and bursicon are crucial for ecdysis and post-ecdysis behaviors^4,8,9,19,40,41^. RNAi studies in *T. molitor* also confirmed essential roles of ETH, EH, CCAP and bursicon and their receptors in the ecdysis sequence although there are some substantial species-specific differences between the aforementioned model insects^12^. Using electrophysiology techniques in *B. mori* we further demonstrate that PETH and ETH action on their receptors in specific central neurons initiate strictly coordinated activity of downstream peptidergic signaling pathways (Fig. 11). These pathways include kinins and DHs controlling pre-ecdysis I, PDF involved in pre-ecdysis II, AST-CC, CCAP and MIPs that initiate the ecdysis behavior, plus sNPFs and MIPs terminating ecdysis. Potential roles of PDF, AST-CC and sNPFs-MIPs in regulation of specific behavioral phases have been described here for the first time.

Experiments in pharate larvae of *M. sexta* revealed that although the ecdysis circuitry is activated ∼10–15 min after initiation of pre-ecdysis, these animals switch to ecdysis with a considerable delay after 40–60 min^30,42^. We hypothesized that the onset of ecdysis is controlled by a balance between excitatory and inhibitory inputs in the CNS. Using ligation experiments, an inhibitory input responsible for the delay of ecdysis onset was localized in the SG and TG1-3^30^. Although cellular sources and factors of this input have not been determined, it is tempting to speculate that a group of unidentified ETHR-B neurons in the SG and TG1-3 may be responsible for inhibition of the ecdysis onset. Since ETHR-B shows higher sensitivity to ETH compared to ETHR-A^7,13,16,35^, we propose that initial low ETH levels first inhibit the ecdysis circuitry via activation of more sensitive inhibitory ETHR-B neurons, whereas increased ETH levels activate less sensitive ETHR-A neurons that mediate ecdysis initiation. This model could explain timing of the switch from pre-ecdysis to ecdysis, with the same ligand providing both excitatory and inhibitory inputs to ecdysis-activating circuits. Acceleration of the ecdysis onset after silencing of specific MIP neurons in *D. melanogaster* suggests possible inhibitory inputs that accounts for delayed switch to the ecdysis behavior^19^.

**Figure 13:**
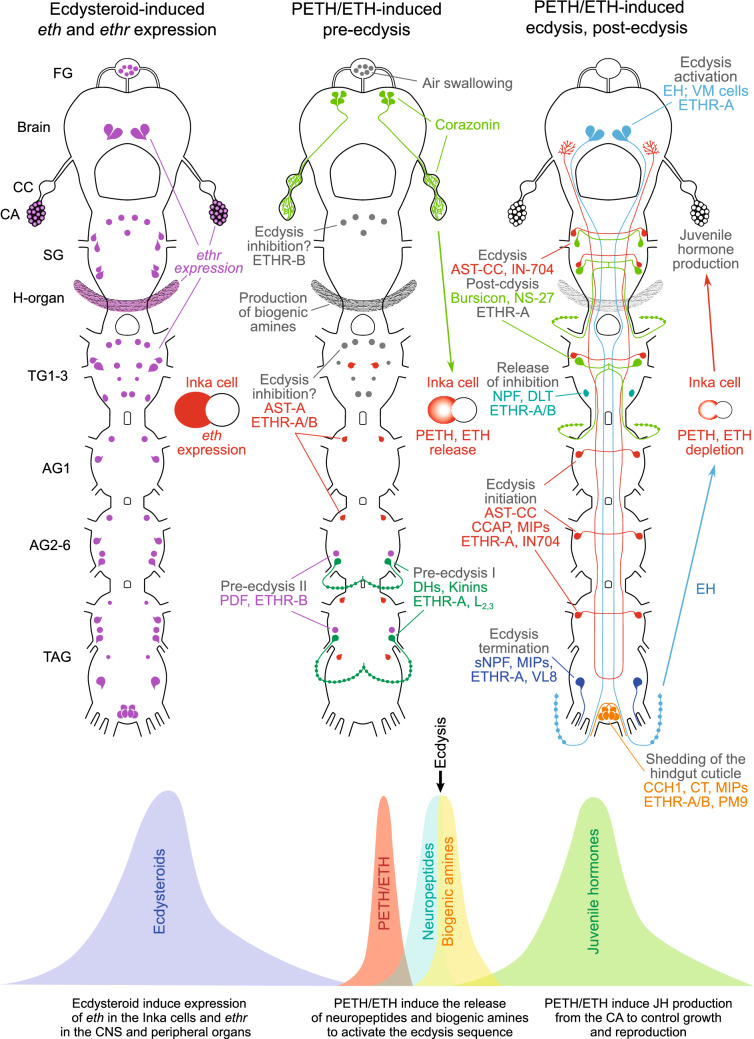
Model for regulation of PETH/ETH expression, release and action. Increased ecdysteroid levels induce expression of *eth* in Inka cells and *ethr* in the CNS, CA, H-organ, epidermis, Malpighian tubules and other organs. Ecdysteroid decline provides signal to release corazonin from the brain-CC-CA complex that elicits secretion of PETH/ETH from Inka cells. PETH/ETH action on multiple targets in the CNS results in initiation of the consecutive phases of the ecdysis sequence. PETH/ETH activation of ETHR-A neurons producing kinins/DHs leads to initiation of pre-ecdysis I, while ETHR-B neurons producing PDF-like peptide control pre-ecdysis II. About 30 min into pre-ecdysis, increased levels of PETH/ETH activate the ecdysis network which is composed of ETHR-A cells that produce EH in the brain and segmental neurons NS-27 and IN-704 expressing AST-CC, CCAP, MIPs and bursicon in the ventral nerve cord. However, the ecdysis onset is delayed by aminergic inhibitory ETHR-B neurons in the SG and TG1-3 and ETHR-A/B neurons producing AST-A in the AG1-8. After 1 h of pre-ecdysis, segmental interneurons DLT1-3 producing NPF suppress the descending inhibition and evoke central release of AST-CC, CCAP, MIPs and bursicon to initiate the ecdysis behavior. Simultaneously, a mixture of neuropeptides CCH1, CT and MIPs produced by ETHR-A/B neurons PM9 control proctodeal contractions to shed the inner cuticle lining the hindgut. Immediately after the old cuticle is shed, the ecdysis behavior is terminated by the central release of sNPF and MIPs from ETHR-A neurons VL8 in the posterior TAG. ETH/EH-activated network of ETHR-A neurons (NS-27 and IN-704) producing bursicon and other factors control post-ecdysis processes—cuticle plasticization, expansion, sclerotization and pigmentation. PETH/ETH action on its receptors in peripheral organs include air swallowing by the FG, production of JH by the CA, production of biogenic amines from the H-organ and regulation of water balance by MT cells on surface of the Malpighian tubules.

A cluster of posterior PM9 cells in the TAG coexpressing both ETHRs and neuropeptides CT, MIPs and CCH1 forms a complex axonal network on muscle surface along the posterior proctodeum. This implies that ETH-activated PM9 neurons release these neuropeptides to regulate contractions essential for shedding of the cuticle lining the hindgut. This hypothesis is indirectly supported by a potent contraction activity of CT and MIPs produced by a neighboring cluster of MAN9 neurons^28^. Proposed model describing roles of different neuropeptides in regulation of different phases of the ecdysis sequence in *B. mori* is schematically depicted in Fig. 13.

### Possible ETHR roles in peripheral organs

Although the expression of both ETHR subtypes in the CNS is well established in several unrelated species^7,8,12,13,16,21,35^, the information regarding ETHR expression and function in other peripheral organs is rather scarce. Previous comprehensive RT-qPCR analysis of *B. mori* GPCRs in early pharate and feeding larvae on day 2 revealed strong expression of ETHR-A in the CC-CA complex, while moderate-weak mRNA levels were detected in the brain, epidermis, midgut and gonads. ETHR-B was only detected in the CC-CA^20^. Our RT-qPCR data confirmed expression of both receptor isoforms in the CNS (with attached H-organ and CC-CA), epidermis, posterior gut and gonads, but surprisingly the highest transcript levels were observed in the Malpighian tubules (Fig. 12b). The discrepancies between these studies could be explained by the fact that different larval stages were used for the analysis [20, this study]. Our data also revealed prolonged ETHR expression in the CNS (Fig. 12a), while expression of ETHRs in peripheral organs is usually restricted to late pharate stages just several hours prior to ecdysis (Fig. 12b–e; Daubnerová, Žitňan, unpublished).

We used ISH to determine exact sites of ETHRs expression in peripheral organs. In the CC-CA complex ISH revealed strong ETHR-A and ETHR-B expression only in the CA, indicating that these endocrine glands are a direct target of ETH (Figs. 2a, 6a). Indeed, allatotropic function of ETH was confirmed by recent studies in the adult mosquito *A. aegypti*, and flies *D. melanogaster* and *B. dorsalis.* In adult females of these species ETH controls JH production by the CA which is essential for development of ovaries and normal reproduction^22–24^. Moreover, ETH appears to stimulate ovulation by its direct action on octopaminergic neurons in the female reproductive tract of *D. melanogaster*^43^. The physiological role of ETH signaling in regulation of JH biosynthesis in other developmental stages has not been elucidated so far, but strong expression of ETHRs in the CA of all pharate stages suggests that ETH action on these receptors could result in stimulation of JH production at each ecdysis. Available data showing transient JH peaks that follow each larval and pupal ecdyses^44,45^ support this hypothesis. Increased JH levels after each larval ecdysis promote growth of juvenile stages and suppress metamorphosis^44,45^. The putative allatotropic role of ETH in these stages, however, requires more investigation.

Detection of ETHR in the FG, H-organ, epidermis, gonads, gut and Malpighian tubules indicates enormous functional diversity of ETH signaling. Presence of ETHRs in the FG is probably associated with activation of the foregut motor programs required for air swallowing during ecdysis as observed in the desert locust *Schistocerca gregaria* and the moth *M. sexta*^46–49^. In vitro experiments in the locust demonstrated that direct action of PETH and ETH on the FG elicits foregut motor pattern known to participate in regulation of the ecdysis sequence^26^. Since ETHR neurons in the FG do not seem to produce any known neuropeptide^50^, activity of these cells is likely mediated by other biologically active compounds.

Increased levels of ETHR transcripts in the Malpighian tubules found in this study (Fig. 13) were also observed in pharate larvae of *B. dorsalis*^35^ and suggest a possible role of ETH in regulation of water balance during and after ecdysis. Using ISH we were able to identify MT cells on surface of the Malpighian tubules that represent novel putative targets for ETH. Strong ETHR expression in the H-organ is likewise intriguing. This large organ is located on dorsal surface of the nerve cord between SG and TG1. Originally, it has been described in larval and pupal stages of several moth species^51,52^ and in nymphs of locusts^53^, but its function is enigmatic. Although it may resemble neurohemal transverse nerves attached to the posterior side of each ventral ganglion, it apparently does not contain any known neuropeptides (this study) or axonal fibers^54^. Our preliminary data indicate that it produces biogenic amines (Koči, Park, Žitňan, unpublished). ETHR expression in small elongated cells of the H-organ may provide hints to elucidate its function. Our attempts to identify specific targets expressing ETHRs in the epidermis, gonads and gut failed due to the high background or unspecific staining.

Although very little is known about mode of action and function of ETH signaling in the peripheral organs, the recent data suggest that it is not restricted to activation of neurons in the CNS. Strong expression of ETHRs in various types of cells and organs implies that ETH may activate multiple peripheral targets to exert functions essential for the next developmental stage. Increased ecdysteroid levels apparently induce expression of ETH and its receptors several hours or days prior to ecdysis^6,13,36,55^, while ecdysteroid decline and consequent release of ETH is required for successful ecdysis and production of JH^4,22–24^. In a line of the established axis (ecdysteroids-ETH-JH), it seems plausible to propose that ETH participates in regulation of feeding, development, water balance and reproduction by a direct action on various organs expressing ETHRs. Hypothetical participation of peripheral organs in regulation of physiological events associated with the ecdysis sequence is described in the proposed model (Fig. 13).

## Materials and methods

### Experimental animals

In this study we used a polyvoltine strain N4 of the silkworm *B. mori*. Larvae were reared on fresh mulberry leaves or a Silk Mate 2 M powder-type artificial diet (Nosan Corporation, Life-Tech Dept., Japan) at 25 °C under 16 h:8 h light:dark photoperiod.

### Cell culture and transfection

Pharmacological characterization of ETHR-A (BNGR-A6-A) and ETHR-B (BNGR-A6-B) receptors were carried out in the Chinese hamster ovary (CHO-K1) cells. For expression in CHO-K1 cells, the entire open reading frame for each ETHR was inserted into pcDNA3.1 ( +) vector (Invitrogen, Carlsbad, CA, USA) with a mammalian Kozak translation initiation sequence incorporated at 5´ end and confirmed by DNA sequencing prior to use. The primers used to clone BNGR-A6-A and BNGR-A6-B are listed in Supplementary Table S1 online. For the assay, each receptor was expressed together with the bioluminescent calcium-sensitive reporter aequorin and chimeric Gqs alpha subunit protein that couples receptor activation to the phospholipase C and intracellular Ca^2+^ mobilization. Plasmids for codon-optimized aequorin and chimeric G proteins were described previously^56,57^. CHO cells were cultured as a monolayer in 10 cm tissue culture dishes in Dulbecco´s modified eagle medium nutrient mixture F-12 Ham (DMEM/F12) (PAN-Biotech GmbH, Germany) supplemented with 10% heat-inactivated fetal bovine serum (Sigma-Aldrich, St Louis, MO, USA), 100 U/mL penicillin and 100 μg/mL streptomycin (Thermo Fisher Scientific, MA, USA) in a humidified atmosphere of 5% CO_2_ at 37 °C. For transient transfection, CHO-K1 cells were grown in 10 cm tissue culture dishes to 60–80% confluence and plasmid DNA was transfected into cells using FuGene HD (Promega, Madison, WI, USA), according to the manufacturer’s instructions. The total amount of DNA was 10 μg per dish, with equimolar ratio of all co-transfected plasmids. Cells were allowed to grow for 24–48 h (37 °C, 5% CO_2_) and used for the assay.

### Calcium mobilization GPCR assay

The procedures used for the receptor assays were described previously^16^. Briefly, the transfected cells were detached using phosphate buffered saline (PBS; 137 mM NaCl, 2,6 mM KCl, 8,1 mM Na_2_HPO_4_ 0,44 mM KH_2_PO_4_) with 0.2% EDTA (pH 8.0), collected in the assay medium (phenol red free DMEM/F12 with l-glutamine and 15 mM HEPES (Thermo Fisher Scientific, MA, USA) supplemented with 0.1% BSA (Sigma-Aldrich, St Louis, MO, USA) and 1% penicillin/streptomycin. Cells were centrifuged for 3 min at 1,000 rpm at RT and the pellet resuspended in the fresh assay medium. Coelenterazine H (Promega, Madison, WI, USA) was added to final concentration of 5 μM and cells were maintained in suspension with gentle stirring for 3 h at RT in the dark. All peptides used in the study were dissolved in the assay medium and a series of various concentrations were loaded in triplicates into a white 96-well plate (Sigma-Aldrich, St Louis, MO, USA). Following cell application to a well, emitted luminescence corresponding to the ligand-induced intracellular Ca^2+^ release was monitored in 0.5 s intervals for 20 s using the GloMax-Multi Detection System (Promega, Madison, WI, USA). As a negative control, wells containing the assay medium without ligands was included in each row to correct specific cell responses. Wells containing ATP at a final concentration of 50 µM served as a positive control and 100 µM digitonin was used to measure the total receptor-independent cellular Ca^2+^ response. All experiments were replicated three times and collected output data calculated and further analyzed in Excel (Microsoft, Redmond, WA, USA) and GraphPad Prism 6 software (GraphPad Software Inc.). The concentration-dependent response curves, as well as the corresponding EC_50_ values were calculated using the Prism software. Synthetic peptides used in the assay are shown in Supplementary Table S2 online.

### In situ hybridization

Preparation of probes and wholemount ISH procedure was previously described in detail^58^. Briefly, we used digoxigenin-labeled single-stranded DNA (ssDNA) probes prepared by asymmetric PCR using cDNA from the CNS of 5th instar larvae and PCR Dig Probe Synthesis Kit (Roche, Mannheim, Germany). To distinguish ETHR-A and ETHR-B expression, reverse primers specific for each splice variant were used to synthesize antisense probes, while sense probes synthesized with forward primers served as negative controls. The same procedure was used to generate all other probes utilized in this study. All primer sequences are listed in Supplementary Table S1 online. For the wholemount ISH procedure, the CNS and peripheral organs were dissected in the physiological saline (140 mM NaCl, 5 mM KCl, 1 mM MgCl_2_, 5 mM CaCl_2_, 4 mM NaHCO_3_, 5 mM HEPES, pH 7.2), fixed in 4% paraformaldehyde, washed with PBS-Tween 20 (PBST), treated with Proteinase K and incubated in a hybridizing solution containing Dig-labelled probes overnight at 48 °C. The tissues were then incubated with alkaline phosphatase-labeled anti-Dig antibody overnight and stained with BCIP/NBT (Roche, Mannheim, Germany). Expression of each transcript was examined in at least five samples dissected from pharate larvae, pupae or adults. Stained tissues were either mounted in glycerol or subjected to immunohistochemistry (see below). ISH staining was observed and photographed under Eclipse 600 microscope with Coolpix 990 camera (Nikon, Tokyo, Japan).

### Immunohistochemistry and antibodies

Procedures for IHC with various antibodies was described previously^28,58^. A list of primary antibodies used in this study is shown in Supplementary Table S3 online. To generate mouse polyclonal antibodies against CCH1, antigenic peptide (CGGCQAYGHVCYGGHamide) was synthesized by Anygen Co (Gwangju, Korea), conjugated to maleimide-activated mariculture keyhole limpet hemocyanin (mcKLH; Thermo Fisher Scientific, MA, USA) and injected three times into mice at two week intervals. The specificity of immunostaining was confirmed by preabsorption of each antibody with its antigen and by ISH using probes specific for the respective neuropeptide transcript as described^28^. A mixture of primary antibodies produced by mice, guinea pigs or rabbits was used for detection of neuropeptides in neurons expressing ETHRs in the CNS. Bound primary antibodies were detected with a mixture of multiple labeling grade secondary antibodies: Alexa Fluor 488-labeled donkey anti-rabbit IgG, Alexa Fluor 594-labeled donkey anti-mouse IgG and Alexa Fluor 647-labeled donkey anti-guinea pig IgG (Jackson ImmunoResearch, Suffolk, UK), diluted 1:1,000. Whole pharate 2-3^rd^ instar larvae were used for staining of peptidergic innervation of peripheral tissues. Anesthetized larvae were cut with spring scissors on the dorsal side along the heart, flattened and pinned in a Sylgard dish (Dow Corning Corporation, Midland, MI, USA), and fixed in 4% paraformaldehyde for 1 h. Fixed larvae were then transferred to Eppendorf tubes and processed for IHC as described^28^. Stained preparations were mounted in glycerol and scanned using TCS SPE-II confocal system (Leica Microsystems, Germany) with 488, 532 and 635 nm lasers for excitation. Scanned images and schematic drawings were processed and labelled using Image J, and Adobe Creative Cloud (Photoshop and Illustrator).

### Electrophysiology and surgical procedures

Specific roles of various neuropeptides produced by the ETHR neurons were determined using electrophysiology technique in the isolated intact or desheathed CNS. Sharp forceps were used to remove the neurolema on the dorsal or ventral side of selected ganglia under physiological saline (see above) and dorsal or ventral nerves of the abdominal ganglia (AG1-7) were attached to plastic suction electrodes. A mixture or individual neuropeptides were then applied in a 300 μl saline bath and induced extracellular motor bursts were recorded using differential AC amplifier 1700 (A-M Systems, Carlsborg, WA, USA) and Axoscope program (Axon Instruments, Union City, CA, USA). Synthetic peptides used in the assay are listed in Supplementary Table S2 online. A mixture of MIPs was adjusted according to its copy number in the precursor: MIP-I (five copies), MIP-II,V (two copies), MIP-III,IV,VI,VII (one copy each)^28^.

### Real-time quantitative PCR (RT-qPCR)

The CNS, fat body, male and female gonads, gut, Malpighian tubules and epidermis from 5th instar larvae were dissected in the saline (see above) and stored in RNAlater (QIAGEN, Hilden, Germany) at 4 °C. For RNA isolation we pooled CNS and peripheral organs from ten and five individuals, respectively. The CNS was dissected from the following stages: feeding 4th instar larvae (∼36 h prior to ecdysis), pharate 5th instar at the head slippage (∼24 h), pigmented new spiracles (∼12 h) and brown mandibles stage (∼2 h), and feeding 5th instar larvae ∼24 h after ecdysis. Peripheral organs were dissected from pharate 5th instar larvae of the brown mandible stage (∼2 h prior to ecdysis). Total RNA was extracted using RNeasy Protect Mini Kit (QIAGEN, Hilden, Germany) and a single stranded cDNA was generated using oligo(dT) primers and Maxima H Minus First Strand cDNA Synthesis Kit (Thermo Fisher Scientific, MA, USA). Transcripts were quantified on a real time PCR machine CFX96 (Bio-Rad, Hercules, CA, USA), by using Xceed qPCR SG Mix (2x) Lo-ROX kit (Institute of Applied Biotechnologies, Praha, Czech Republic). Forward and reverse primers were designed to match the common exon 2 and either ETHR-A specific exon 3a or ETHR-B specific exon 3b. The primer sequences are listed in Supplementary Table S1 online. Transcript levels of analyzed receptors were measured in three technical replicates and normalized to the levels of reference genes RpL3 and Rp49. Three biological replicates were used for each quantitative analysis.

## Supplementary Information

Supplementary Information.

## References

[1] Ewer, J. & Reynolds, S. Neuropeptide control of molting in insects. In *Hormones, Brain and Behavior* (ed. D. W. Pfaff, et al.) 1–92 (Boston, Elsevier Science, 2002).

[2] Webster SG, Keller R, Dircksen H (2012). The CHH superfamily of multifunctional hormones controlling crustacean metabolism, osmoregulation, moulting and reproduction. Gen. Comp. Endocrinol..

[3] Webster SG, Wilcockson DC, Mrinalini Sharp JH (2013). Bursicon and neuropeptide cascades during the ecdysis program of the shore crab Carcinus maenas. Gen. Comp. Endocrinol..

[4] Žitňan, D. & Adams, M. E. Neuroendocrine regulation of ecdysis. *Insect endocrinology* (ed. Gilbert, L. I.) 253–309 (Elsevier, 2012).

[5] Žitňan D, Kingan TG, Hermesman JL, Adams ME (1996). Identification of ecdysis-triggering hormone from an epitracheal endocrine system. Science.

[6] Žitňan D, Ross LS, Žitňanová I, Hermesman JL, Gill SS, Adams ME (1999). Steroid induction of a peptide hormone gene leads to orchestration of a defined behavioral sequence. Neuron.

[7] Kim YJ, Žitňan D, Cho KH, Mizoguchi A, Schooley D, Adams ME (2006). Central peptidergic ensembles associated with organization of an innate behavior. Proc. Natl. Acad. Sci. USA.

[8] Kim YJ, Žitňan D, Galizia CG, Cho KH, Adams ME (2006). A Command chemical triggers an innate behavior by sequential activation of multiple peptidergic ensembles. Curr. Biol..

[9] Diao F, Mena W, Shi J, Park D, Diao F, Taghert P, Ewer J, White BH (2016). The splice isoforms of the *Drosophila* ecdysis triggering hormone receptor have developmentally distinct roles. Genetics.

[10] Žitňan D, Žitňanová I, Spalovská I, Takáč P, Park Y, Adams ME (2003). Conservation of ecdysis-triggering hormone signalling in insects. J. Exp. Biol..

[11] Roller L, Žitňanová I, Dai L, Šimo L, Park Y, Satake H, Tanaka Y, Adams ME, Žitňan D (2010). Ecdysis triggering hormone signaling in arthropods. Peptides.

[12] Arakane Y, Li B, Muthukrishnan S, Beeman RW, Kramer KJ, Park Y (2008). Functional analysis of four neuropeptides, EH, ETH, CCAP and bursicon, and their receptors in adult ecdysis behavior of the red flour beetle *Tribolium castaneum*. Mech. Dev..

[13] Dai L, Adams ME (2009). Ecdysis triggering hormone signaling in the yellow fever mosquito *Aedes aegypti*. Gen. Comp. Endocrinol..

[14] Ewer J, Gammie SC, Truman JW (1997). Control of insect ecdysis by a positive-feedback endocrine system: roles of eclosion hormone and ecdysis triggering hormone. J. Exp. Biol..

[15] Gammie SC, Truman JW (1997). Neuropeptide hierarchies and the activation of sequential motor behaviors in the hawkmoth *Manduca sexta*. J. Neurosci..

[16] Park Y, Kim YJ, Dupriez V, Adams ME (2003). Two subtypes of ecdysis-triggering hormone receptor in *Drosophila melanogaster*. J. Biol. Chem..

[17] Clark AC, del Campo ML, Ewer J (2004). Neuroendocrine control of larval ecdysis behavior in *Drosophila*: complex regulation by partially redundant neuropeptides. J. Neurosci..

[18] Lahr EC, Dean D, Ewer J (2012). Genetic analysis of ecdysis behavior in *Drosophila* reveals partially overlapping functions of two unrelated neuropeptides. J. Neurosci..

[19] Kim DH, Han MR, Lee G, Lee SS, Kim YJ, Adams ME (2015). Rescheduling behavioral subunits of a fixed action pattern by genetic manipulation of peptidergic signaling. PLoS Genet.

[20] Yamanaka N, Yamamoto S, Žitňan D, Watanabe K, Kawada T, Satake H, Kaneko Y, Hiruma K, Tanaka Y, Shinoda T, Kataoka H (2008). Neuropeptide receptor transcriptome reveals unidentified neuroendocrine pathways. PLoS ONE.

[21] Lenaerts C, Cools D, Verdonck R, Verbakel L, Vanden Broeck J, Marchal E (2017). The ecdysis triggering hormone system is essential for successful moulting of a major hemimetabolous pest insect *Schistocerca gregaria*. Sci. Rep..

[22] Areiza M, Nouzova M, Rivera-Perez C, Noriega FG (2014). Ecdysis triggering hormone ensures proper timing of juvenile hormone biosynthesis in pharate adult mosquitoes *Insect Biochem*. Mol. Biol..

[23] Meiselman M, Lee SS, Tran R-T, Dai H, Ding Y, Rivera-Perez C, Wijesekeraf TP, Dauwalderf B, Noriega FG, Adams ME (2017). Endocrine network essential for reproductive success in *Drosophila melanogaste*r. Proc. Natl. Acad. Sci. USA.

[24] Shi Y, Liu T-Y, Jiang H-B, Liu X-Q, Dou W, Park Y, Smagghe G, Wang J-J (2019). The ecdysis triggering hormone system, via ETH/ETHR-B, is essential for successful reproduction of a major pest insect, *Bactrocera dorsalis* (Hendel). Front. Physiol..

[25] Ayali A (2004). The insect frontal ganglion and stomatogastric pattern generator networks. Neurosignals.

[26] Zilberstein Y, Ewer J, Ayali A (2006). Neuromodulation of the locust frontal ganglion during the moult: a novel role for insect ecdysis peptides. J. Exp. Biol..

[27] Shimomura M, Minami H, Suetsugu Y, Ohyanagi H, Satoh C, Antonio B, Nagamura Y, Kadono-Okuda K, Kajiwara H, Sezutsu H, Nagaraju J, Goldsmith MR, Xia Q, Yamamoto K, Mita K (2009). KAIKObase: an integrated silkworm genome database and data mining tool. BMC Genomics.

[28] Čižmár D, Roller L, Pillerová M, Sláma K, Žitňan D (2019). Multiple neuropeptides produced by sex-specific neurons control activity of the male accessory glands and gonoducts in the silkworm *Bombyx mori*. Sci. Rep..

[29] Veenstra JA (2009). Allatostatin C and its paralog allatostatin double C: the arthropod somatostatins. Insect Biochem. Mol. Biol..

[30] Žitňan D, Adams ME (2000). Excitatory and inhibitory roles of central ganglia in initiation of the insect ecdysis behavioural sequence. J. Exp. Biol..

[31] Žitňan D, Hollar L, Spalovská I, Takáč P, Žitňanová I, Gill SS, Adams ME (2002). Molecular cloning and function of ecdysis-triggering hormones in the silkworm *Bombyx mori*. J. Exp. Biol..

[32] Gammie SC, Truman JW (1999). Eclosion hormone provides a link between ecdysis-triggering hormone and crustacean cardioactive peptide in the neuroendocrine cascade that controls ecdysis behaviour. J. Exp. Biol..

[33] Davis NT, Blackburn MB, Golubeva EG, Hildebrand JG (2003). Localization of myoinhibitory peptide immunoreactivity in *Manduca sexta* and *Bombyx mori*, with indications that the peptide has a role in molting and ecdysis. J. Exp. Biol..

[34] Iversen A, Cazzamali G, Williamson M, Hauser F, Grimmelikhuijzen CJ (2002). Molecular identification of the first insect ecdysis triggering hormone receptors. Biochem. Biophys. Res. Commun..

[35] Shi Y, Jiang HB, Gui SH, Liu XQ, Pei YX, Xu L (2017). Ecdysis triggering hormone signaling (ETH/ETHR-A) is required for the larva-larva ecdysis in *Bactrocera dorsalis* (Diptera: Tephritidae). Front. Physiol..

[36] Žitňanová I, Adams ME, Žitňan D (2001). Dual ecdysteroid action on the epitracheal glands and central nervous system preceding ecdysis of *Manduca sexta*. J. Exp. Biol..

[37] Sakurai S, Kaya M, Satake S (1998). Hemolymph ecdysteroid titer and ecdysteroid-dependent developmental events in the last-larval stadium of the silkworm, *Bombyx mori*: role of low ecdysteroid titer in larval-pupal metamorphosis and a reappraisal of the head critical period. J. Insect. Physiol..

[38] Mizoguchi A, Ohashi Y, Hosoda K, Ishibashi J, Kataoka H (2001). Developmental profile of the changes in the prothoracicotropic hormone titer in hemolymph of the silkworm *Bombyx mori*: correlation with ecdysteroid secretion. Insect Biochem. Mol. Biol..

[39] Dai L, Dewey EM, Žitňan D, Luo CW, Honegger HW, Adams ME (2008). Identification, developmental expression, and functions of bursicon in the tobacco hawkmoth *Manduca sexta*. J. Comp. Neurol..

[40] Peabody NC, Diao F, Luan H, Wang H, Dewey EM, Honegger HW, White BH (2008). Bursicon functions within the *Drosophila* CNS to modulate wing expansion behavior, hormone secretion, and cell death. J. Neurosci..

[41] Kruger E, Mena W, Lahr EC, Johnson EC, Ewer J (2015). Genetic analysis of eclosion hormone action during *Drosophila* larval ecdysis. Development.

[42] Fuse M, Truman JW (2002). Modulation of ecdysis in the moth *Manduca sexta*: the roles of the suboesophageal and thoracic ganglia. J. Exp. Biol..

[43] Meiselman MR, Kingan TG, Adams ME (2018). Stress-induced reproductive arrest in *Drosophila* occurs through ETH deficiency-mediated suppression of oogenesis and ovulation. BMC Biol..

[44] Kinjoh T, Kaneko Y, Itoyama K, Mita K, Hiruma K, Shinoda T (2007). Control of juvenile hormone biosynthesis in *Bombyx mori*: cloning of the enzymes in the mevalonate pathway and assessment of their developmental expression in the corpora allata. Insect Biochem. Mol. Biol..

[45] Furuta K, Ichikawa A, Murata M, Kuwano E, Shinoda T, Shiotsuki T (2013). Determination by LC-MS of juvenile hormone titers in hemolymph of the silkworm *Bombyx mori*. Biosci. Biotechnol. Biochem..

[46] Bell, R. A. Role of the frontal ganglion in lepidopterous insects in I*nsect Neurochemistry and Neurophysiology* (ed. Borkovec, A. B. & Gelman, D. B.) 321 -324 (Clifton, NJ: Humana Press, 1986).

[47] Bestman JE, Miles CI, Booker R (1997). Neural and behavioral changes associated with larval molts in the moth *Manduca sexta*. Soc. Neurosci. Abstr..

[48] Miles CI, Booker R (1998). The role of the frontal ganglion in the feeding and eclosion behavior of the moth *Manduca sexta*. J. Exp. Biol..

[49] Zilberstein Y, Ayali A (2002). The role of the frontal ganglion in locust feeding and moulting-related behaviours. J. Exp. Biol..

[50] Roller L, Čižmár D, Gáliková Z, Bednár B, Daubnerová I, Žitňan D (2016). Molecular cloning, expression and identification of the promoter regulatory region for the neuropeptide trissin in the nervous system of the silkmoth *Bombyx mori*. Cell Tiss. Res..

[51] Abou-Halawa S, Sláma K (1986). A neurohemal organ in the prothorax of Lepidoptera. Ent. Bohemoslov..

[52] Bhargawa S, Sláma K (1987). Growth of the prothoracic H-organ in larvae of *Galleria*. J. Insect Physiol..

[53] Mutun S, Ober A (1998). The localization and structure of a neurohemal H-organ in *Acrida bicolor* (Thunberg) and *Locusta migratoria* (Linneaus) (Orthoptera). Tr. J. Zool..

[54] Birkenbeil H (1997). Electron microscopic observations on the H-organ of Lepidoptera. Eur. J. Entomol..

[55] Žitňan D, Kim YJ, Žitňanová I, Roller L, Adams ME (2007). Complex steroid-peptide-receptor cascade controls insect ecdysis. Gen. Comp. Endocrinol..

[56] Yapici N, Kim Y-J, Ribeiro C, Dickson BJ (2008). A receptor that mediates the post-mating switch in *Drosophila* reproductive behaviour. Nature.

[57] Kim YJ, Bartalska K, Audsley N, Yamanaka N, Yapici N, Lee J-Y, Kim Y-C, Markovic M, Isaac E, Tanaka Y, Dickson BJ (2010). MIPs are ancestral ligands for the sex peptide receptor. Proc. Natl. Acad. Sci. USA.

[58] Bednár B, Roller L, Čižmár D, Mitrová D, Žitňan D (2017). Developmental and sex-specific differences in expression of neuropeptides derived from allatotropin gene in the silkmoth *Bombyx mori*. Cell Tissue Res..

