# Supplementary material for: Identification and function of ETH receptor networks in the silkworm Bombyx mori
Source: Sci Rep. 2021 Jun 3;11:11693. doi: 10.1038/s41598-021-91022-8 (PMC8175484; doi:10.1038/s41598-021-91022-8)
Supplement: Supplementary file 1 — Supplementary Information. [file 41598_2021_91022_MOESM1_ESM.pdf]

**Identification and function of ETH receptor networks in the silkworm *Bombyx mori***

Ivana Daubnerová[1], Ladislav Roller[1], Honoo Satake[2], Chen Zhang[3], Young-Joon Kim[3], Dušan Žitňan[1]*

[1]Institute of Zoology, Slovak Academy of Sciences, Dúbravská cesta 9, 84506, Bratislava, Slovakia
[2]Bioorganic Research Institute, Suntory Foundation for Life Sciences, Kyoto, Japan
[3]School of Life Sciences, Gwangju Institute of Science and Technology (GIST), Oryongdong, Buk-gu, Gwangju 61005, Republic of Korea

*Corresponding author: Dušan Žitňan, dusan.zitnan@savba.sk

## SUPPLEMENTARY INFORMATION

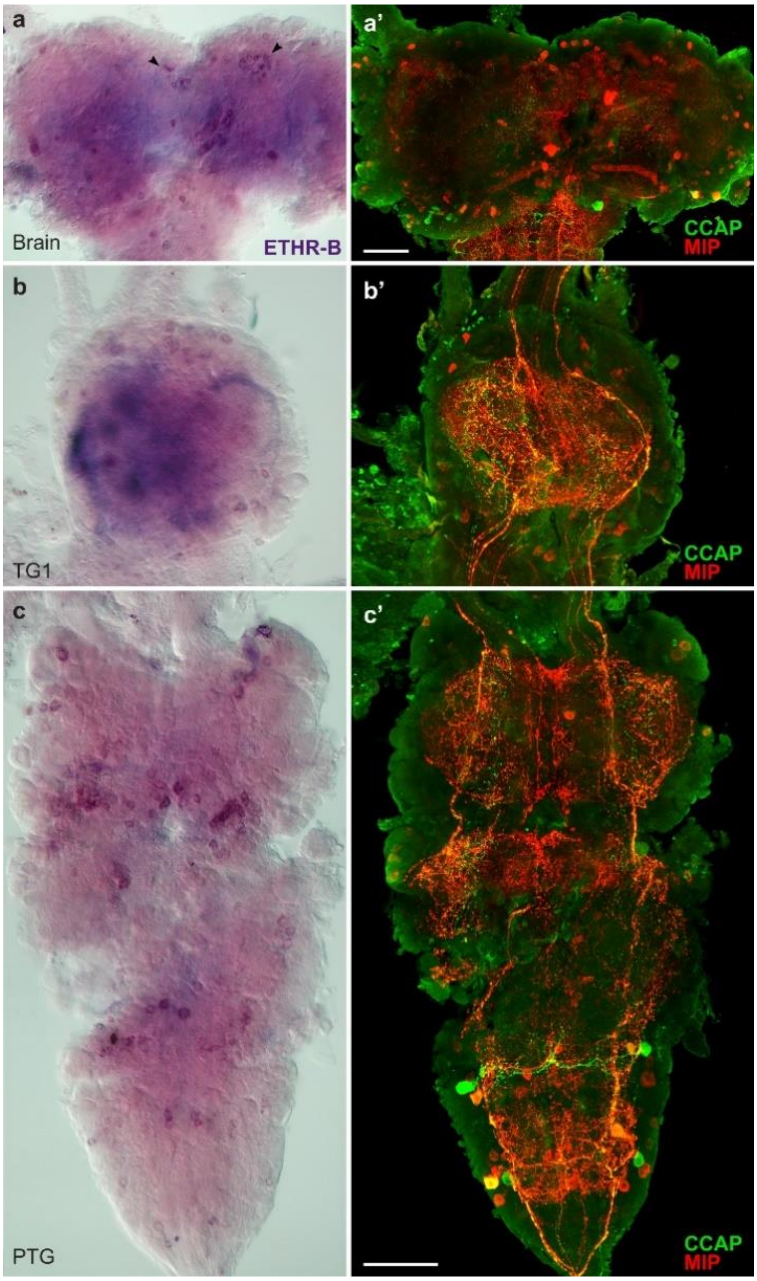


**Supplementary figure S1**. ETHR-B expression in the CNS of pharate adults. (**a-c**) ETHR-B transcript detected by ISH in 20-30 small neurons in each lateral protocerebrum (arrowheads)-and in numerous cells in the brain, SG, TG1 and PTG. (**a´-c´**) None of these neurons were stained with antibodies to CCAP (green) and MIP (red).

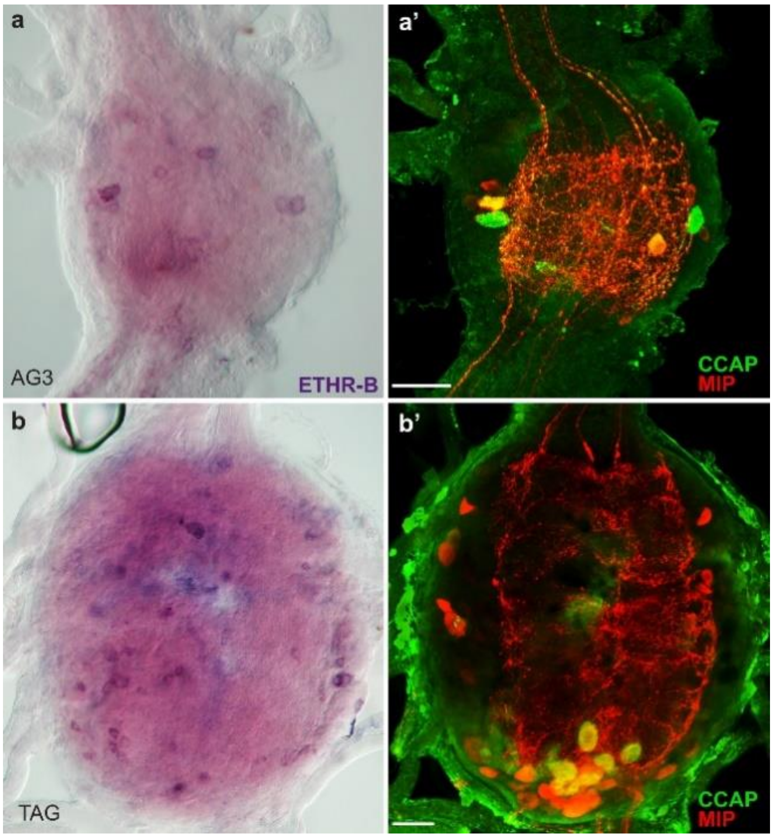


**Supplementary figure S2.** ETHR-B expression detected by ISH in the AG3 and TAG of pharate adults (**a-b**) does not overlap with neurons producing CCAP (green) and MIPs (red) (**a´-b´**).

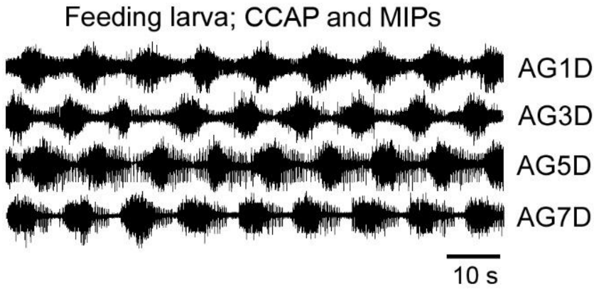


**Supplementary figure S3.** Normal ecdysis bursts induced in 1-3 min by CCAP and MIPs in the desheathed CNS of the feeding 5$^{th}$ instar larva on day 1.

**Primers Used for *In Situ* Hybridization Constructs**

| Gene | Accession | Forward primer | Reverse primer |
|---|---|---|---|
| ETHR-A | NM_001134269.1 | 5′-CACAATCATCATCCTCCTCT-3′ | 5′-GACTTCTGAAAAGCCACAAC-3′ |
| ETHR-B | NM_001172266.1 | 5′-CCGCCTTATTCTTCATCCTG-3′ | 5′-ATCTATTTGCGCGTCTTCGT-3′ |
| AST-CC | fmgV41d19f | 5′-GACGCAACGCCTCATACTAA-3′ | 5′-ACGCAGCAATTTACCGAAAC-3′ |
| sNPF | AB330419 | 5′-GAATTCGTGTAACCCCATCG-3′ | 5′-ATCTAGGTTGCGACGAGAGG-3′ |
| DDC | AF372836.1 | 5′-TTGGATTGGTTGGGTCAAAT-3′ | 5′-GGAGAAACGCGAGCAAATAG-3′ |
| CCH1 | XM_004930480 | 5′-CAACGTCGACTAGGCAGGAC-3′ | 5′-CCGGTCATTAATTGCCAGAT-3′ |

**Primers for RT-qPCR**

| Gene | Accession | Forward primer | Reverse primer |
|---|---|---|---|
| ETHR-A | NM_001134269.1 | 5′-TACGTGTGCACGAAGACCAG-3′ | 5′- ATTCACGACGGTCCCATTG-3′ |
| ETHR-B | NM_001172266.1 | 5′-TACGTGTGCACGAAGACCAG-3′ | 5′- GCACCTCAGTTCCATCTTCG-3′ |
| Rp49 | NM_001098282.1 | 5′-CAGGCGGTTCAAGGGTCAATAC-3′ | 5′-TGCTGGGCTCTTTCCACGA-3′ |
| RpL3 | AB024901.1 | 5′-AGCACCCCGTCATGGGTCTA-3′ | 5′-TGCGTCCAAGCTCATCCTGC-3′ |

**Primers used for GPCR assay constructs**

| Gene | Primer name | Primer sequence |
|---|---|---|
| ETHR-A/B | (NheI)ETHRa-L | 5′- AATGCTAGCCACCATGATTTCAACGATTAACTACAC -3′ |
| ETHR-A | (ApaI)ETHRa-R | 5′- TATGGGCCCTTAAACGAAACTCTCTGGG -3′ |
| ETHR-B | (ApaI)ETHRb –R | 5′- TATGGGCCCTTAAACGAAACTTTCTTTAGTAT -3′ |

**Supplementary Table S1.** List of primers used in this study.

| Peptides | Amino acid sequence |
|---|---|
| Bom PETH | S**FIK**P**N.NVPR**Va |
| Bom ETH | **SN**EAISPFDQG**M**MGYV**IK**T**NKN**I**PR**Ma |
| Trc ETH1 | ENYVL**KA**A**KNVPR**Ia |
| Trc ETH2 | **SN**TNKNTNIDE**M**GKF**F**M**KA**S**K**S**VPR**Ia |
| Pea ETH1 | SEYDNF**F**L**KA**S**K**S**VPR**Ia |
| Drm Hug | pQLQSNGEPAYRVRT**PR**La |
| Bom Kinin-I | N**FSPWG**a |
| Bom Kinin-II | VR**FSPWG**a |
| Mas DH30 | **S**FSVNPAVDILQHRYMEKV**A**QN**NRNFLN**RVa |
| Mas DH41 | RMPSLSIDLPM**S**VLRQKLSLEKERKVHALR**A**AA**NRNFLN**DIa |
| Bom PDF | NADLINSLLALPKDMNDAa |
| CCAP | PFCNAFTGCa |
| Bom MIP-I | **AWQDLNSAW**a |
| Bom MIP-II | G**WQDLNSAW**a |
| Bom MIP-III | APEK**W**AAFHGS**W**a |
| Bom MIP-IV | G**W**N**D**IS**S**V**W**a |
| Bom MIP-V | **AWQD**MS**SAW**a |
| Bom MIP-VI | **AW**SA**L**HGT**W**a |
| Bom MIP-VII | S**WQDLNS**V**W**a |
| Bom ATL-II | GTPTFKSPTVGI**A**R**DF**a |
| Bom ATL-III | MKFTPKIGLMV**A**H**DF**a |
| Bom sNPF-I | S**PS**R**RLRF**a |
| Bom sNPF-I | T**P.**V**RLRF**a |
| Bom sNPF-I | A**PS**M**RLRF**a |
| Bom RFa-I | SAI**D**R**S**M**IRF**a |
| Bom RFa-II | SA**SFVRF**a |
| Bom RFa-III | **D**P**SFIRF**a |
| Bom AST-CC | GQSNNNRGRVLRCFFNAVTCF-OH |

**Supplementary Table S2.** List of peptides used in the study. Lowercase "a" indicates C-terminal amidation, "OH" indicates C-terminal carboxyl group, "pQ" at the N terminus indicates pyroglutamate. Conserved amino acids of related peptides are in bold. PETH and ETH, pre-ecdysis and ecdysis triggering hormones; Hug, Hugin; DH30 and DH41, CRF-like diuretic hormones 30 and 41; PDF, pigment dispersing factor; CCAP, crustacean cardioactive peptide; MIP I-VII, myoinhibitory peptides I-VII; ATL-II,III; allatotropin-like peptides II, III; AST-CC, allatostatin CC; sNPF, short neuropeptide F; RFa, FIRFamide. Prefixes for peptides indicate species of origin: *Bom*, *Bombyx mori*; Mas, *Manduca sexta*; Drm, *Drosophila melanogaster*; Trc, *Tribolium castaneum*; Pea, *Periplaneta americana.*

| Primary antibody to | Type | Dilution | Reference |
|---|---|---|---|
| Allatostatin A (AST-A) | Mm | 1:200 | [59] |
| Allatostatin C (AST-C) | Rb | 1:1,000 | [60] |
| Allatotropin (AT) | Rb | 1:2,000 | [60] |
| Bursicon | Gp | 1:2,000 | [3] |
| CCHamide 1 (CCH1) | Mp | 1:2,000 | This study |
| Calcitonin-like diuretic hormone (CT) | Rb | 1:1,000 | [61] |
| Corazonin | Rb | 1:4,000 | [62] |
| CRF-like diuretic hormone (DH) | Rb | 1:2,000 | [60] |
| Crustacean cardioactive peptide (CCAP) | Rb | 1:1,000 | [63] |
| Eclosion hormone (EH) | Rb | 1:1,000 | [31] |
| ITP | Rb | 1:10,000 | [64] |
| ITPL | Rb | 1:10,000 | [64] |
| Kinin | Rb | 1:1,000 | [65] |
| Myoinhibitory peptide (MIP) | Mm | 1:1,000 | [7] |
| Myosupressin (BMS) | Mm | 1:2,000 | [66] |
| Orcokinin (ORC) | Mp | 1:4,000 | [67] |
| Pigment dispersing hormone (PDF) | Rb | 1:3,000 | [68] |
| RFamide | Rb | 1:3,000 | [69] |
| RFamide | Gp | 1:3,000 | [69] |
| Tachykinin (TK) | Rb | 1.1,000 | [70] |

**Supplementary Table S3**. List of primary antibodies used in the study. Gp, guinea pig polyclonal; Mm, mouse monoclonal; Mp, mouse polyclonal; Rb, rabbit polyclonal

## Supplementary references


59. Stay, B., Chan, K. K. & Woodhead, A. P. Allatostatin-immunoreactive neurons projecting to the corpora allata of adult *Diploptera punctata*. *Cell Tiss. Res.* **270**, 15–23 (1992).

60. Žitňan, D., Kingan, T. G., Kramer, S. J. & Beckage, N. E. Accumulation of neuropeptides in the cerebral neurosecretory system of *Manduca sexta* larvae parasitized by the braconid wasp *Cotesia congregata*. *J. Comp. Neurol.* **356**, 83–100 (1995).

61. Park, D., Veenstra, J. A., Park, J. H. & Taghert, P. H. Mapping peptidergic cells in *Drosophila*: where DIMM fits in. *PLoS ONE* **3**, e1896 (2008).

62. Roller L, Tanaka Y, Tanaka S. Corazonin and corazonin-like substances in the central nervous system of the pterygote and apterygote insects. *Cell Tissue Res* 312:393–406 (2003).

63. Stangier, J., Hilbich, C., Dircksen, H. & Keller, R. Distribution of a novel cardioactive neuropeptide (CCAP) in the nervous system of the shore crab *Carcinus maenas*. *Peptides* **9,** 795–800 (1988).

64. Dai L, Žitňan D, Adams ME. Strategic expression of ion transport peptide gene products in central and peripheral neurons of insects. *J Comp Neurol* 500:353-367 (2007).

65. Nässel, D. R., Cantera, R. & Karlsson, A. Neurons in the cockroach nervous system reacting with antisera to the neuropeptide leucokinin I. *J. Comp. Neurol.* **322**, 45–67 (1992).

66. Yamanaka, N., Hua, Y. J., Mizoguchi, A., Watanabe, K., Niwa, R., Tanaka, Y. & Kataoka, H. Identification of novel prothoracicostatic hormone and its receptor in the silkworm, *Bombyx mori*. *J. Biol. Chem*. **280**, 14684–14690 (2005).

67. Yamanaka N, Roller L, Žitňan D, Satake H, Mizoguchi A, Kataoka H, Tanaka Y. Bombyx orcokinins are brain-gut peptides involved in the neuronal regulation of ecdysteroidogenesis. *J Comp Neurol* 519: 238-246 (2011).

68. Persson, M. G. S., Eklund, M. B., Dircksen, H., Muren, J. R. & Nässel, D. R. Pigment-dispersing factor in the locust abdominal ganglia may have roles as circulating neurohormone and central neuromodulator. *J. Neurobiol*. **48**, 19–41 (2001).

69. Grimmelikhuijzen, C. J. P. & Spencer, A. N. FMRFamide immunoreactivity in the nervous system of the medusa *Polyorchis penicillatus*. *J. Comp. Neurol.* **230**, 361–371 (1984).

70. Schoofs L, Vanden Broeck J, De Loof A. The myotropic peptides of Locusta migratoria: structures, distribution, functions and receptors. *Insect Biochem Mol Biol* 23:859-881 (1993).
